# Long-term independent use of an intracortical brain–computer interface for speech and cursor control

**DOI:** 10.1038/s41591-026-04414-6

**Published:** 2026-06-15

**Authors:** Nicholas S. Card, Tyler Singer-Clark, Hamza Peracha, Carrina Iacobacci, Xianda Hou, Maitreyee Wairagkar, Zachery Fogg, Elena C. Offenberg, Leigh R. Hochberg, Sergey D. Stavisky, David M. Brandman

**Affiliations:** 1 Department of Neurological Surgery, University of California, Davis, Davis, CA USA; 2 Department of Neurology and Neurosurgery, University Medical Center Utrecht Brain Center, Utrecht University, Utrecht, the Netherlands; 3 School of Engineering and Carney Institute for Brain Science, Brown University, Providence, RI USA; 4 VA Center for Neurorestoration and Neurotechnology, Rehabilitation Research Development and Translation Service, Department of Veterans Affairs, Providence, RI USA; 5 Department of Neurology, Massachusetts General Hospital, Boston, MA USA

**Keywords:** Brain-machine interface, Motor cortex, Premotor cortex

## Abstract

Brain–computer interfaces (BCIs) can provide naturalistic communication and digital access to people with severe paralysis by decoding neural activity associated with attempted speech and movement. Recent work has demonstrated highly accurate intracortical BCIs for speech and cursor control, but two critical capabilities needed for practical viability were unmet: independent at-home operation without researcher assistance and reliable long-term performance supporting accurate speech and cursor decoding. Here we demonstrate the independent and near-daily use of a multimodal BCI with novel brain-to-text speech and computer cursor decoders by a man with paralysis and severe dysarthria due to amyotrophic lateral sclerosis. Over nearly 2 years, the participant used the BCI for more than 3,800 h at home with no researchers present to maintain rich interpersonal communication with his family and friends, independently control his personal computer and sustain full-time employment—despite being paralyzed. He communicated 183,060 sentences—totaling 1,960,163 words—at an average rate of 56 words per minute. He labeled 92% of sentences as being decoded at least mostly correctly. In formal quantifications of performance where he was asked to say words presented on a screen, attempted speech was consistently decoded with more than 99% word accuracy (125,000 word vocabulary). The participant also used the speech BCI as keyboard input and the cursor BCI as mouse input to control his personal computer, enabling him to send text messages and emails and to browse the internet. These results demonstrate that intracortical BCIs have the potential to support independent use in the home, marking a critical step toward practical assistive technology for people with severe motor impairment.

## Abstract (web summary)

An automated intracortical brain–computer interface, used at home with no researcher intervention, provides long-term and accurate restoration of speech-based communication and cursor-based computer usage in a person with severe dysarthria due to amyotrophic lateral sclerosis.

## Main

The loss of the ability to speak and use digital devices profoundly impacts the independence and quality of life of people with severe motor impairments caused by conditions such as amyotrophic lateral sclerosis (ALS) or brainstem stroke^1–3^. Although a variety of augmentative and assistive communication technologies exist to support communication and computer use for people with paralysis, these tools are often slow, fatiguing or unreliable or require frequent intervention from care partners trained in their use^4^. Recent progress in brain–computer interfaces (BCIs) has shown a promising alternative pathway toward restoring naturalistic communication and digital access, by decoding neural activity during attempted speech or movement into words or digital actions^5–13^.

Intracortical BCIs, which record neural signals at the resolution of action potentials, have enabled the highest-performance decoding to date of cursor^9^, handwriting^6^, typing^13^ and speech^5,12^. Intracortical BCIs have traditionally focused on decoding either speech or hand movement-based control, often relying on separate cortical regions and independent computational architectures. Recent studies have shown that speech-related activity in the motor speech cortex can be decoded into text with high speed and accuracy, enabling brain-to-text communication at near-conversational rates^5,7,10^. Other work has demonstrated that neural signals related to intended arm and hand movements can drive high-performance cursor control^9,14–17^, and one recent study has shown that both capabilities can be decoded from the speech motor cortex^18^. However, most prior intracortical BCIs have addressed speech or cursor control in isolation, required frequent recalibration to maintain accuracy and/or required researcher oversight to don and doff the system. Moreover, existing BCIs have typically been evaluated in short-term research settings, without a demonstration of extended high performance over many months of independent use.

These limitations highlight two major barriers to the clinical translation of intracortical BCI technology. First, the transition from a scientific demonstration to a practical communication device requires users (and their care partners) to be able to operate the system independently in their homes, without daily support from researchers or technicians. Second, neural recording sensors and decoding performance must remain stable over long durations despite the neural signal variability associated with chronic implants^19–21^. Ideally, little-to-no time should be spent asking the user to interrupt their use to recalibrate the decoder.

Here, we report the long-term, independent use of a multimodal intracortical BCI that enables both brain-to-text speech and computer cursor control. This study builds upon the core brain-to-text decoding capability of ref. ^5^ and ventral motor cortex cursor control of ref. ^18^ and adds new decoding architectures with higher accuracy and stability, thousands of hours of independent BCI usage over several months and a refined BCI user interface with additional features that enhance the efficacy of independent communication and digital access.

With care partner assistance largely restricted to donning and doffing the hardware and initializing the software, a man with paralysis and severe dysarthria due to ALS used the system in his home nearly every day for 19 months, accumulating more than 3,800 h of independent use. The system incorporated novel decoding architectures for both speech and cursor control, as well as multiple software features that enabled independent use and continuous decoder fine-tuning. For speech, we developed a transformer-based brain-to-text decoder that outperformed prior recurrent neural network (RNN)-based models^5^, requiring little-to-no daily calibration and achieving a new state-of-the-art word accuracy of 99.2% in a prompted word copy task (125,000-word vocabulary). For cursor control, we implemented an RNN-based decoder that matched or exceeded the performance of linear models, also with reduced calibration requirements. Using this system, the participant communicated more than 180,000 sentences at conversational speeds and used the speech and cursor decoders together to independently operate his personal computer—sending messages, browsing the internet, participating in video calls and maintaining full-time employment—despite being paralyzed.

These results demonstrate that intracortical BCI systems, equipped with advanced decoding algorithms, can now support rich, independent digital and in-person communication in real-world settings. These results are a substantial step toward delivering a practical assistive technology for people with severe speech and motor impairments.

## Results

### Overview

**Fig. 1:**
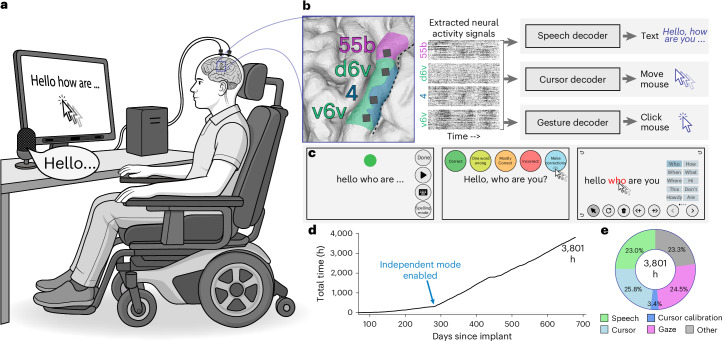
Independent use of the multimodal intracortical BCI. **a**, A schematic of the participant using the multimodal intracortical BCI to control their personal computer. As the participant tried to speak, his neural activity was decoded into words on a screen. He could also control his computer by attempting to move or squeeze his hand. The system also integrated eye tracking to enable the participant to select on-screen buttons by looking at them for a short time. The participant could switch between gaze-based and neural cursor-based control of the user interface by selecting his preference through the custom-made GUI. **b**, Left: the locations of four 64-microelectrode arrays in the participant’s dominant ventral precentral gyrus. Arrays were placed in areas 55b, dorsal 6v (d6v), 4 and ventral 6v (v6v). The dashed line is the dominant central sulcus. Each electrode records activity from individual neurons and local field potentials. Recorded neural signals were processed into binned sequences of neural features (middle) and inputted to speech, cursor or gesture decoders to decode the user’s intended words, cursor movements or clicks (right). **c**, User interface examples during speech decoding (left), sentence rating (middle) and sentence correction (right). **d**, Cumulative hours of system usage over time. Independent use began at post-implant day 281. **e**, The time distribution of how the system was used across 3,801 h sourced from all personal use sessions. ‘Other’ refers to idle time where the BCI system was active, but the participant was not using it.

A 45-year-old man with paralysis and severe dysarthria due to ALS (‘T15’) enrolled in the BrainGate2 clinical trial (ClinicalTrials.gov number, NCT00912041) in 2023. In total, four microelectrode arrays (64 electrodes each) were surgically placed in T15’s ventral precentral gyrus (speech motor cortex) to record intracortical neural activity via percutaneous wired connections (Fig. 1a and Fig. 1b, left). Neural signals (Fig. 1b, center) were processed by three parallel neural decoders for real-time translation of T15’s neural activity into intended words, cursor movements and click gestures (Fig. 1b, right). A continuously running ‘brain-to-text’ decoder detected and decoded attempted speech into the most likely word sequences. Two additional decoders—activated by a gaze-controlled toggle—translated right-hand motor imagery into two-dimensional cursor control and discrete mouse clicks. Details of decoder architectures and training procedures are provided in Methods.

The BCI system in T15’s home was a set of networked research computers^22^ mounted on a mobile cart, located in his bedroom or living room. T15’s care partners were trained to safely connect the neural recording equipment and power on the computer system, which was then automatically initialized through custom software. This allowed T15 to operate the BCI without assistance from any member of the research team. T15 and his care partners reported that daily setup took approximately 20 min, after which he used the system continuously for up to 19 h without additional assistance. Once initialized, T15 operated the system independently using neural decoding, supplemented by eye gaze tracking to facilitate user-interface control. The speech decoder software ran continuously in the background and displayed decoded words on screen in real time (Fig. 1c, left and Supplementary Video 1). After each utterance, T15 was given the option to make corrections to the decoded text through a custom user interface via gaze or cursor input (Fig. 1c, right). He then rated decoding accuracy with the following options: ‘correct’, ‘one word wrong’, ‘mostly correct’ or ‘incorrect’ (Fig. 1c, middle, and Supplementary Video 2). Examples of sentences with each rating are in Table 1. T15 was instructed to rate decoded sentence accuracy strictly, such that he would only mark a sentence as ‘correct’ if it was exactly what he tried to say, even down to plurality (for example, ‘cat’ versus ‘cats’).

**Table 1:** Example decoded sentences for each accuracy rating

Rating	Decoded sentence
Correct	he wants a work plan before he leaves to go back to australia
Correct	step number six is the mastery of the skill and the ability to navigate emergency situations
Correct	can you turn on the air conditioning i am getting hot
One word wrong	thank you and i will have this later
One word wrong	and the computer is taking that attention and turning it into words on the screen in front of me
One word wrong	if not no worries but i will need to know today because it will require us to modify our classes to be ready in time
Mostly correct	thank you i am expecting other news i am simply assessing my other needs
Mostly correct	very decision because you will see so many jokes
Mostly correct	he was also creating a video game for killer whales
Incorrect	we were to understand or figure out in that moment
Incorrect	that is not what i am talking about i want to get access to the premium waters for the anna doll
Incorrect	it is more of a concern for men in here i was just looking for a general category that a button or a light one or i am a big deal

Selected personal use transcripts shared with participant’s permission.

We previously quantified T15’s natural speech and effective communication rates with his preferred augmentative and assistive communication device^5^. Rather than relying on his existing communication strategies, T15 chose the BCI as his primary mode of communication and computer interface. He used it to converse with family, friends, colleagues and clinicians—in person, over video calls and through digital applications such as email and text messaging. He also used it for both professional and recreational computer access. Before post-implant day 281, usage was limited to 2–4 weekly sessions requiring a research assistant (author C.I.) to oversee setup and use (3.7 h d^−1^ average). After the investigational device exemption (IDE) was modified allowing his care partners to don and doff the system without the scientific team being present on day 281, T15 and his care partners could initiate system use independently at their home whenever they wanted, resulting in a marked increase in usage to 9.5 h d^−1^ average (Fig. 1d). As of 22.6 months after the implant surgery, he had accumulated more than 3,800 h of BCI use, and he used the BCI on 444/653 days (Fig. 1e). The participant used the BCI at his own discretion. Days where the BCI was not used may have happened for a variety of private reasons (for example, the participant was traveling or simply chose not to use the system).

### Speech decoding

**Extended Data Fig. 1:**
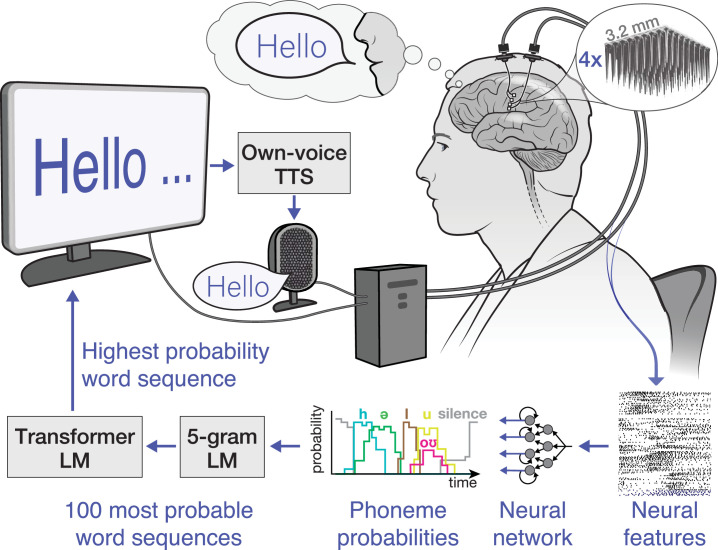
Brain-to-text decoding pipeline. Neural signals are recorded from the speech motor cortex via four 64-electrode ‘Utah’ arrays as the participant attempts to speak. The neural activity is processed in real time into neural features, which are inputted into a neural network to predict the most likely phonemes that the participant is trying to say every 80 ms. Phoneme probability sequences are inputted into a multi-stage language model to identify the most likely word sequence being spoken. Predicted words are then displayed as text on a display in real time, and at the end of a sentence they are read aloud by a text to speech (TS) model that was optimized to sound like the user’s pre-ALS voice.

**Extended Data Fig. 2:**
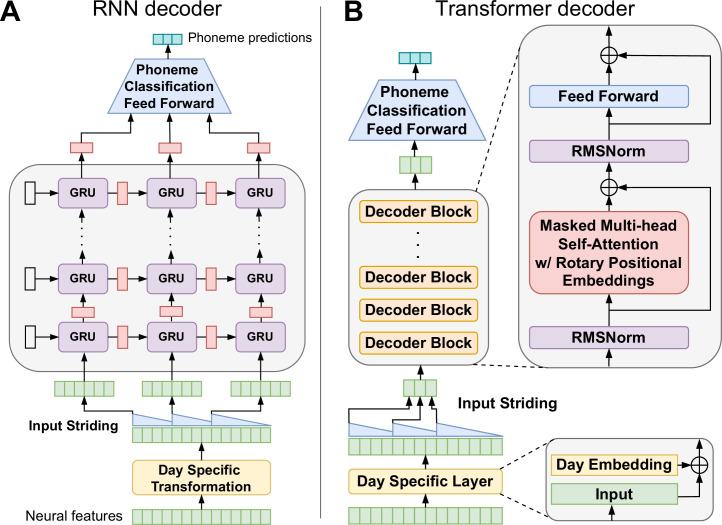
Brain-to-text decoder architectures. (**a**) A recurrent neural network (RNN) based neural network that predicts the most likely phoneme being spoken from sequences of neural features. Neural features are inputted to the model and then undergo a day-specific transformation with the goal of aligning nonstationary neural data from separate days into a common subspace. Aligned features are then rearranged into overlapping time windows (‘input striding’) before being passed to a series of GRU layers. Finally, a feedforward layer converts the output to the probability that any English phoneme is being spoken. This model was trained with connectionist temporal classification (CTC) loss, which automatically handles alignment between neural data and phoneme labels. (**b**) A transformer-based neural network that predicts the most likely phoneme being spoken from sequences of neural features. The inputs, outputs, and loss function of this model are identical to the RNN model in (**a**), but the transformer-based internal architecture is different. We implemented multi-head self-attention and applied causal masking so that the model can be used in a streaming application. We used Rotary Positional Embeddings for positional encodings and RMSNorm for normalizing activations before attention layers and feedforward layers.

After implant day 281, T15 used the brain-to-text decoder to communicate independently on a near-daily basis. The decoding pipeline (Extended Data Fig. 1) converted neural features into English phoneme probabilities every 80 ms via a neural network (Extended Data Fig. 2), followed by language modeling to generate the most likely word sequences drawn from a vocabulary of more than 125,000 English words. The decoder was continuously recalibrated in the background to compensate for slow shifts in neural activity and maintain high decoding accuracy. The output was shown on screen after each word and was optionally synthesized at the end of a sentence using a text-to-speech system trained to match T15’s pre-ALS voice (Supplementary Video 5). Over the course of the study, we iteratively improved the decoder architecture—starting from the approach described in ref. ^5^—to increase accuracy, reduce explicit calibration requirements and improve robustness to signal variability (Extended Data Fig. 2). T15 could enable an optional ‘privacy mode’ when he did not want data recorded (Supplementary Video 7); these epochs were not (could not) be further analyzed.

**Fig. 2:**
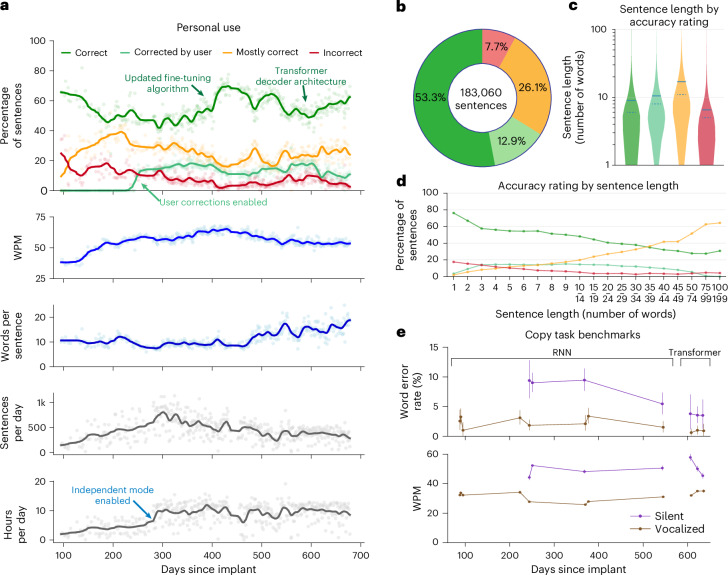
Speech decoding. **a**, The ‘personal use’ speech decoding usage and accuracy statistics by day. The first subplot shows the percentage of sentences that the participant reported to be (1) immediately correct, (2) initially not correct but corrected through the user interface, (3) mostly correct or (4) incorrect. Subplots 2–5 show the daily average rate of communication (WPM), average words per sentence, total sentences communicated and total hours of system use. To provide additional context, text and arrows on subplots indicate when substantive changes were made to the BCI system. **b**, The distribution of self-reported sentence correctness among 183,060 sentences sourced from all personal use sessions. Color legend is the same as the top plot in **a**. All self-reported sentence accuracy ratings were made at the participant’s discretion. **c**, Cumulative distributions of sentence lengths as a function of participant-rated decoding accuracy. Solid horizontal blue lines are means, and dashed lines are medians. **d**, The sentence correctness rating probability displayed as a function of sentence length. **e**, The word error rate (top) and speaking rate (bottom) in periodic copy task benchmark sessions using silent (violet) and vocalized (brown) speaking strategies. Each session had 50–200 evaluation sentences, dependent upon the amount of available time and the participant’s energy. Circles denote per-session mean word error rate or WPM values, and vertical lines denote 95% confidence intervals. The final three benchmark sessions used the transformer-based decoder, and all prior sessions used the RNN-based decoder.

**Extended Data Fig. 3:**
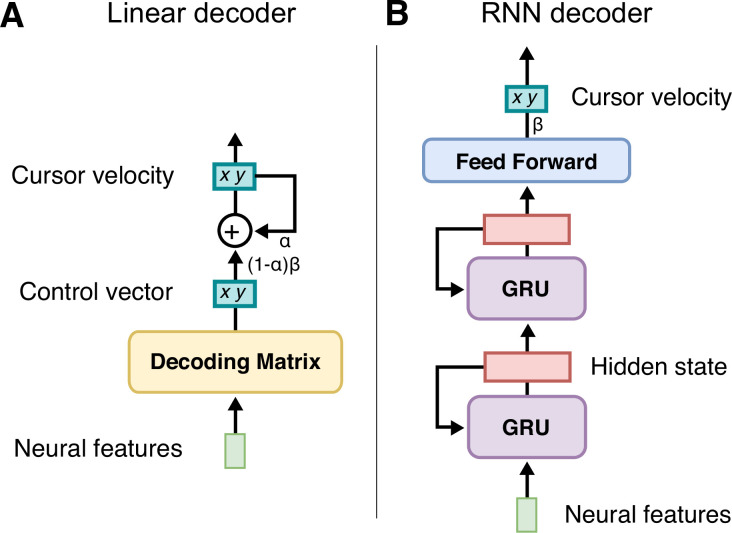
Cursor decoder architectures. (**a**) A linear velocity decoder with smoothing, for decoding intended cursor velocity from neural features. For each 10 time bin, a decoding matrix projects a vector of neural features into a 2-D control vector (the ‘dimensionality reduction’ step). This is averaged with the previous time bin’s predicted velocity (the ‘smoothing’ step) where α sets the balance between the current control vector and the previous velocity and β modulates the overall speed. (**b**) A recurrent neural network (RNN) based neural network for decoding intended cursor velocity from neural features. For each 10 ms time bin, a vector of neural features is inputted to a 2-layer GRU model. A feedforward layer transforms the output of the GRU into a 2-D velocity vector. β modulates the overall speed. The hidden state from each GRU layer is passed back into the model for the following time bin.

**Extended Data Fig. 4:**
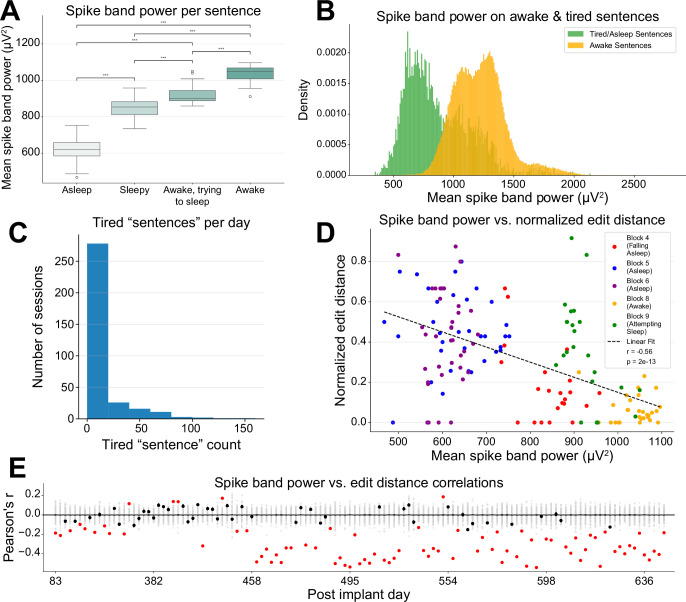
The participant’s level of alertness affects neural signals and speech decoding accuracy. (**a**) Relationship between mean spike band power and putative alertness level. Neural data were recorded in a structured research session while the participant was observed to be asleep, sleepy, awake but trying to sleep, and awake. Spike band power varied significantly with alertness level (ANOVA with post hoc comparisons; ***p<0.001). (**b**) Distributions of spike band power during independent BCI use while the participant was alert versus tired/asleep. Each datum represents the mean spike band power for a sentence; when T15 was nominally asleep, the speech decoder occasionally falsely detected attempted speech and decoded nonsense ‘sentences’, and we use those epochs alongside alert speaking epochs for analysis here. Data from all personal use sessions where eye tracking data was available are aggregated, resulting in 139,364 alert sentences and 3,714 tired/asleep ‘sentences’. Alertness level was inferred using eye tracking data: if no eye tracking data was available for 2 min or more, alertness was categorized as tired/sleeping. (**c**) Number of daily ‘sentence’ trials where the participant was tired or sleeping during independent use. 37.0% of days had at least 5 sentences that were categorized as tired. (**d**) Relationship between mean spike band power and speech decoding accuracy for one example day. Here, speech decoding accuracy is quantified as the edit distance between the predicted phoneme sequence output from the RNN/Transformer neural decoder and the predicted phoneme sequence output from the language model – higher values indicate worse speech decoding. Points are individual trials, colored according to the participant’s estimated alertness level. A linear trendline was fit to the data, which had a significantly negative slope, indicating that speech decoding tended to be better on trials with higher average spike band power values which corresponded to when the participant was estimated to be more alert. (**e**) Linear correlation values between mean spike band powers and edit distance per trial across days of independent use that included 5 or more tired trials. Chance correlations were estimated with 100 shuffled datasets’ values and shown in light gray. Significant correlations (p < 0.01) are marked in red.

**Extended Data Fig. 5:**
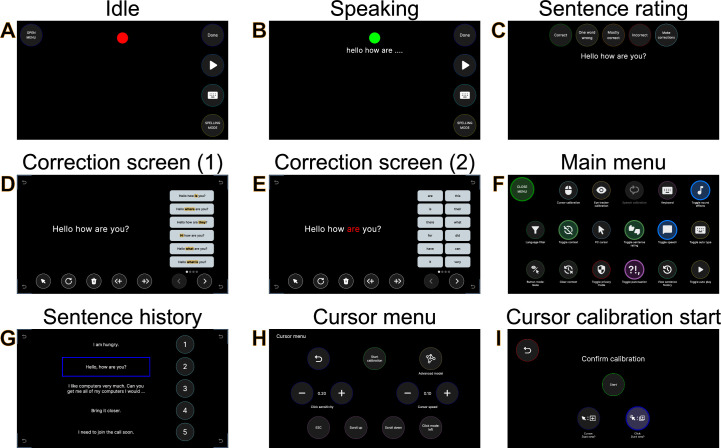
BCI system user interface. (**a**) Graphical user interface (GUI) during idle epochs, where the participant is not speaking. On-screen buttons can be selected with gaze or cursor control. The ‘OPEN MENU’ button opens the menu screen (**f**). The ‘DONE’ button manually stops speech decoding when pressed. The button with a play symbol (▶) plays the most recently decoded sentence aloud using text-to-speech, and the keyboard button pastes that sentence into the active text field on the participant’s personal computer. The ‘SPELLING MODE’ button triggers spelling mode, where the language model vocabulary is restricted to the 26 English letters rather than the normal ~125,000-word vocabulary (Video 6). (**b**) GUI during speaking epochs. When speech is detected, the center circle turns green, decoded words appear on-screen in real time, and the menu button is hidden. When the user is done speaking, they can press the ‘DONE’ button to manually end the sentence or they can wait for a 6-second timeout, after which the sentence will be automatically ended. (**c**) GUI during sentence rating, which occurs at the end of every sentence unless the feature is turned off in the menu by the user. The user can choose to rate the accuracy and move on, or to make corrections to the sentence using the interfaces in (**d**) and (**e**). (**d**) GUI for making corrections to incorrectly decoded sentences. Here, no incorrect word is selected so alternative sentences are shown on the right side, with word differences highlighted in yellow. Corner revert-icon buttons allow the user to exit this screen. The circular buttons on the bottom, from left to right, are: toggle between gaze control and cursor control, refresh word suggestions, delete selected word, insert new word to the left of selected word, insert new word to the right of selected word, page forward, page back. (**e**) Same as (**d**), except now an incorrect word has been selected by the user (red font) and alternative words are shown in the list on the right. (**f**) Main menu screen. Here, the user can enable or disable various functions or enter submenus by selecting each option. The button in the top left exits the menu back to the idle screen as in (**a**). (**g**) Sentence history screen, entered using the corresponding button in the menu in (**f**). Recently decoded sentences are shown and can be selected for playing through TTS or pasting into the user’s personal computer. (**h**) Cursor menu screen, entered using the corresponding button in the menu in (**f**). Here, the user can toggle between the linear or RNN-based cursor decoder, change cursor speed and click sensitivity, toggle presses of function keys for their personal computer, and enter the cursor calibration game. (**i**) Confirmation screen before the cursor calibration game begins. The user can decide whether to update the cursor decoder, click decoder, or both.

Decoding accuracy was self-rated by T15 after each sentence and remained relatively stable over months (Fig. 2a, top). The ability to make corrections using the custom-built graphical user interface, controlled via gaze or neural cursor (Extended Data Fig. 3), became available at post-implant day 227. We observed that sentence accuracy was influenced by multiple factors, including fatigue (Extended Data Fig. 4), attempted speaking rate (Fig. 2a, subplot 2), sentence length (Fig. 2a, subplot 3, and Fig. 2c,d) and topic. System updates—including new feature implementations, bug fixes and decoder upgrades—also contributed to performance variability (Extended Data Fig. 2). Across 183,060 sentences, 53.3% were decoded completely correctly, 12.9% were corrected by the participant, and 26.1% were mostly correct (Fig. 2b). Of the 23,566 sentences that T15 corrected via the user interface (Extended Data Fig. 5), 12.7% of words (31,557/248,586) were changed during the correction process. The overall trend of decoding performance improved over time, with later months showing a higher proportion of correct sentences (two-sided rank sum test; *P* < 0.001) and increased sentence length (*P* < 0.001; Fig. 2a, subplot 3). We note that T15 often strung multiple sentences together at a time into a single utterance—the longest correctly decoded utterance was 215 words (Fig. 2c,d)—which had the effect of lowering the self-rated accuracies (as an utterance gets longer, the probability of it being decoded 100% correctly decreases).

**Extended Data Fig. 6:**
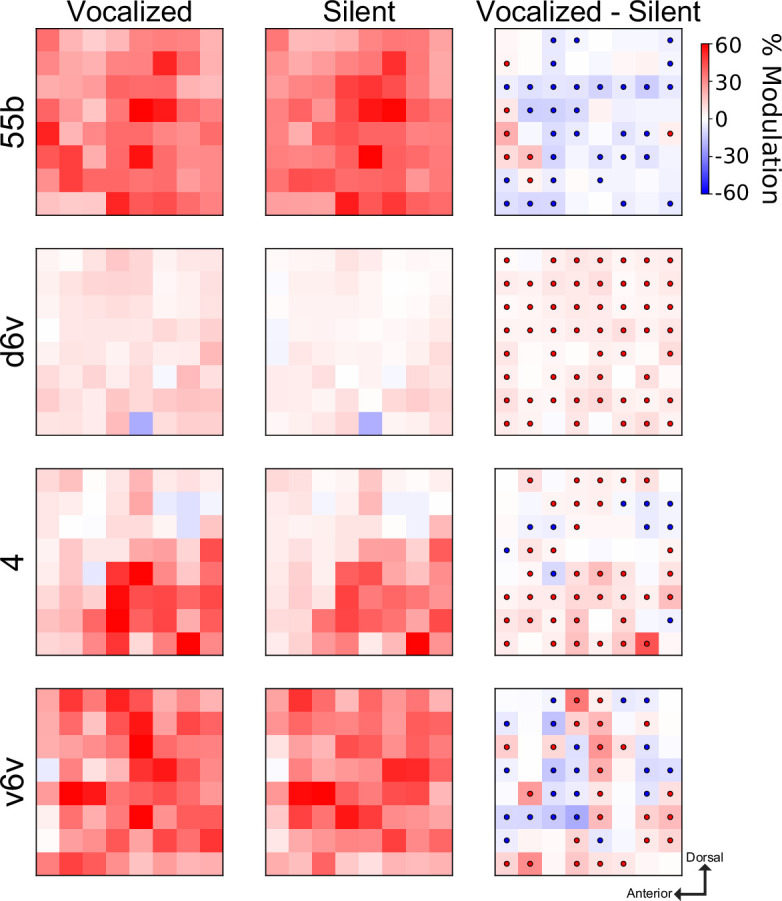
Neural modulation during attempted vocalized or silent speech. Spike band power modulation for each microelectrode on each array during attempted vocalized or silent speech. Data is sourced from 8 research sessions containing both speaking strategies and includes n=1857 trials of attempted vocalized speaking and n=1008 trials of attempted silent speaking. Consistent with Fig. 4c, modulation depth for each electrode is calculated as the average change in spike band power between speaking epochs and non-speaking epochs for each trial. In the third column, a two-sided 2-sample t-test (alpha = 0.05 / 256) is used to compare the distributions of modulation depth for attempted vocalized and attempted silent speaking trials. Red circles indicate that attempted vocalized speaking had significantly more neural modulation than attempted silent speaking. Blue circles indicate that attempted silent speaking had significantly more modulation than attempted vocalized speaking.

We did not instruct T15 to use a particular speaking strategy during personal use of the system; instead, he was encouraged to use whichever approach felt most sustainable, natural and effective. The opportunity to develop a strategy that minimizes user fatigue was especially important, given the known challenges of fatigue in patients with late-stage ALS^23^. Initially, he employed attempted ‘vocalized speech’, producing sound while speaking. Over time, he transitioned to a ‘silent speech’ strategy—making facial muscle movements without phonating—which he noted was less effortful for him. This strategy change was associated with an increase in speaking speed, from ~30 to more than 50 words per minute (WPM; Fig. 2a, subplot 2). To benchmark longitudinal performance of the system, and to quantify the effect of silent versus overt speaking strategies, we periodically asked him to repeat sentences presented on a screen in a copy task (Fig. 2e). During vocalized speech, accuracy exceeded 99% at 30.6 WPM average; with silent speech, accuracy reached 96.5% at 49.7 WPM average. This difference in decoding accuracy may be attributable to differences in neural modulation between the attempted vocalized and silent speaking strategies (Extended Data Fig. 6). Note that personal use and benchmark sessions after post-implant day 600 utilized a more accurate transformer-based phoneme decoding architecture, whereas prior sessions used an RNN-based model (Extended Data Fig. 2).

### Cursor decoding

Although T15’s microelectrode arrays were placed in the ventral precentral gyrus (that is, speech motor cortex), we previously described^18^ how he performed two-dimensional cursor movements and click events using motor imageries (hand or body-part-agnostic) that traditionally are used when decoding point-and-click from dorsal (hand) precentral gyrus. In structured grid task assessments, the system achieved 2.90 ± 0.16 (mean ± s.d.) bits per second of throughput, comparable to performance levels reported with arrays placed in hand motor areas^9,17^.

**Fig. 3:**
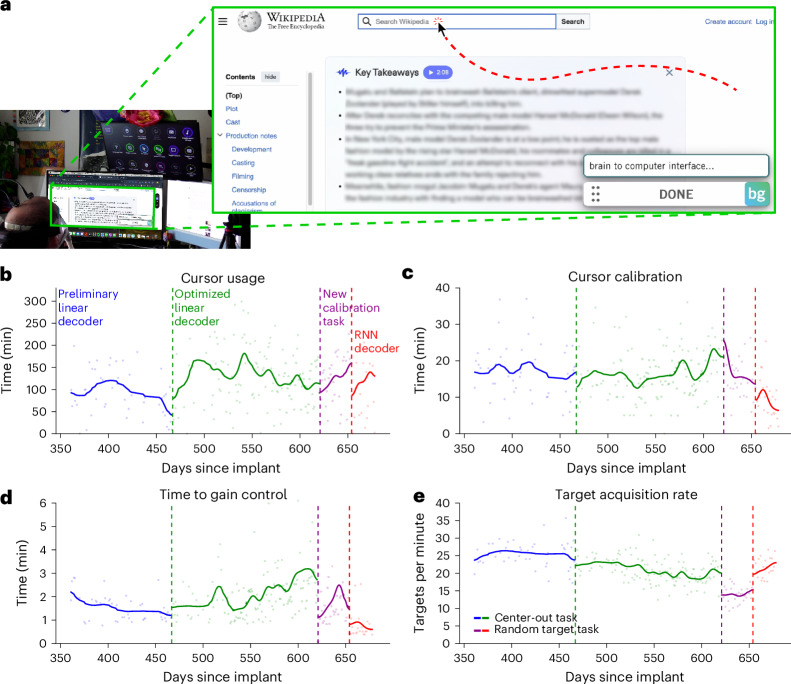
Computer control with the cursor BCI. **a**, A graphic of T15 using the BCI with his personal computer to search Wikipedia for ‘brain to computer interface’. The current decoded words are shown in the bottom right hand of the screen via our custom BG Home software. T15 can move and click the mouse via the cursor decoder to select the search bar. **b**, The amount of time T15 used the cursor BCI to operate his computer. The cursor BCI served as T15’s primary method of computer control. Blue and green data points utilized a linear decoder (preliminary or optimized, respectively) and a center-out-and-back calibration task. Purple data points used the optimized linear decoder with a random target calibration task. Red data points used an RNN-based decoder with a random target calibration task. **c**, The amount of daily calibration time required for the cursor decoder. **d**, The time to gain control of the cursor BCI for the first time each day. T15 maintained the ability to calibrate in a few minutes or less over many months. **e**, The performance of the cursor BCI. T15 maintained the ability to select targets quickly over many months.

Building on the finding of usable neural cursor control, we developed a custom software interface linking the BCI system to T15’s personal computer, enabling him to control the mouse pointer with decoded neural signals (Fig. 3a). By enabling him to ‘copy/paste’ the text decoded by the speech BCI, he could use his computer for a range of daily activities including writing, web browsing and participation in video calls (Supplementary Video 12). Cursor control functionality was added on post-implant day 358, after which T15 used the feature for an average of 121.0 ± 66.9 (mean ± s.d.) minutes per day (Fig. 3b).

T15 configured and toggled cursor control through the BCI’s gaze-based interface, which allowed him to select on-screen buttons to start or stop control, paste recent decoded sentences and issue function key presses (for example, escape key; Extended Data Fig. 5). He could also use the neural cursor to navigate the BCI user interface (Supplementary Video 11), although he typically stuck to gaze-based control there while reserving the neural cursor for his personal computer. Each day, he calibrated the cursor and click decoders via a short calibration routine^17,18^. For calibration, we used a center-out-and-back target acquisition task with fixed target distance and size until day 621, after which we used a more difficult task with varied target positions and smaller target sizes (Methods).

Initially, the cursor velocity was decoded using a linear model, which required an average of 2.0 ± 1.2 min of calibration to gain closed-loop cursor control on a new day (Fig. 3d) and an average of 16.9 ± 7.9 min of total calibration per day (Fig. 3c). On day 654, we replaced the linear model with an RNN-based decoder (Extended Data Fig. 3 and Supplementary Video 3), which significantly reduced calibration time to just 0.8 ± 0.6 min to gain cursor control (rank sum test; *P* < 0.001) and 9.2 ± 5.9 total minutes per day (*P* < 0.001), while maintaining or improving performance (Fig. 3e). This upgrade substantially improved usability, enabling longer, uninterrupted periods of independent computer control.

### Neural signal stability

Speech and cursor decoding performance remained accurate for more than 19 months post-implant, suggesting that the underlying neural signals retained sufficient quality to support high-accuracy decoding. To directly examine the stability of these signals, we analyzed neural activity across the 678-day post-implant period.

**Fig. 4:**
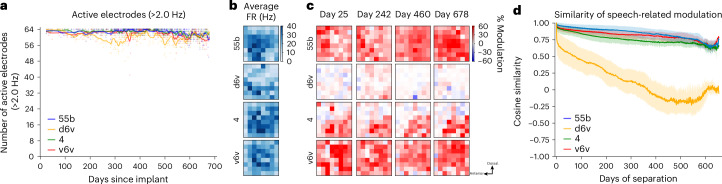
Neural stability. **a**, The number of electrodes per array with firing rates of 2 Hz or greater during speaking epochs, plotted by post-implant day. Dots are individual data points, and lines are Gaussian-smoothed approximations. Colors correspond to the four arrays. **b**, The average firing rate for each electrode during speaking epochs from the entire 444-session dataset. Electrode locations are drawn according to their physical location on each of the four arrays, where up corresponds to dorsal and left corresponds to rostral. **c**, The speech-related neural modulation for each electrode on a given example day, expressed as the percentage change in spike band power between rest epochs and speaking epochs. Each column corresponds to the post-implant day indicated by the column title. Days were chosen to uniformly span the post-implant period (25–678 days post-implant). **d**, The cosine similarity between daily speech-related neural modulation vectors, as a function of the number of days between each pair. Colors correspond to the four arrays. Solid lines are means, and shaded regions represent 1 s.d. around the mean.

**Extended Data Fig. 7:**
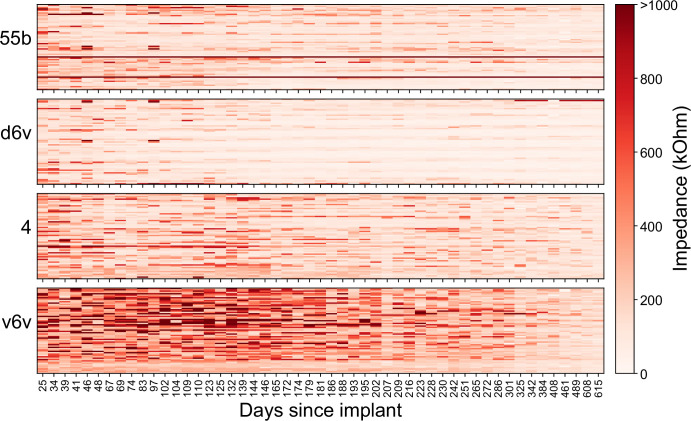
Impedance values over time. Impedance values for each microelectrode on each array were regularly measured through the Blackrock Central software interface. Impedance values for each electrode over time are shown here, grouped by array, according to the color bar.

Multi-unit action potentials were consistently detected on nearly all electrodes throughout the study. More than 90% of electrodes on each 64-electrode array could reliably detect spiking activity at 2 Hz or higher throughout more than 19 months after implant (Fig. 4a). The dorsal 6v (d6v) array occasionally exhibited fewer active spiking electrodes than the other arrays during speaking epochs, consistent with its lower tuning for speech^5^ and stronger tuning for cursor control^18^. Average firing rates for each electrode during speech epochs across the entire dataset are shown in Fig. 4b. Impedance values for each microelectrode on each array were measured on many (but not all) sessions (Extended Data Fig. 7).

To assess the stability of task-relevant neural modulation, we measured the change in spike band power between rest and speech epochs for each electrode on each day. These speech modulation maps appeared qualitatively stable across months (Fig. 4c). We quantified this by computing the cosine similarity between daily neural modulation vectors for each array as a function of time separation (Fig. 4d). Cosine similarities for all arrays—except the d6v array, which did not exhibit much speech-related modulation—remained above 0.6 even for comparisons separated by more than 18 months, demonstrating that task-relevant neural representations remained quite stable for at least 19 months.

## Discussion

This study demonstrates that an intracortical BCI can support long-term, independent communication and computer control by a person with severe paralysis and dysarthria due to ALS. Over a 19-month period, the participant used the system in his home for more than 3,800 h, speaking more than 183,000 sentences, browsing the internet, managing personal and professional communications and maintaining full-time employment. These outcomes establish that a single, speech motor cortex BCI can provide practical, multimodal assistive technology for daily life—enabling both speech and cursor control with high performance and without researcher supervision.

This work successfully addresses two critical gaps that have limited the clinical viability of high-performance intracortical BCIs: the need for researcher-free, at-home operation and the need for sustained decoding performance over long durations. Although previous studies have demonstrated high speech or cursor control accuracy in controlled settings, few have supported independent daily use in a home environment, and none to our knowledge have done so for speech or for both speech and computer control simultaneously. Prior work with electrocorticographic BCIs has shown that independent, at-home use is achievable^24,25^, although these systems relied on click-based decoding, which offers slower communication rates than attempted speech. Whether multielectrode array-based BCIs could support independent and stable long-term use remained previously unknown. Here, the participant and his care partners were able to operate the system independently after an initial training period, and he continued to use it almost every day over nearly 2 years (444/653 days overall, 364/397 days after independent use was enabled).

The system’s stability and usability were made possible by several advances. First, we developed improved architectures for both speech and cursor decoding that reduced calibration time and increased robustness. The speech decoder, built on a transformer-based model, achieved state-of-the-art performance (99.2% word accuracy in copy tasks and 125,000-word vocabulary) while requiring little or no daily explicit recalibration from the user (0–20 prompted calibration sentences at the start of each day at the user’s discretion). The speech decoder’s computational expressiveness^26^ and continuous fine-tuning also helped accommodate neural nonstationarities that have challenged earlier BCI systems with lower channel counts and less flexible algorithms^27^. The cursor decoder, upgraded from a linear model to an RNN, similarly reduced calibration time while preserving or improving performance. Both modalities were decoded from the same neural activity recorded in speech motor cortex, where we found sufficient tuning for both speech-articulatory and hand motor imagery. Second, we implemented software features—such as background decoder calibration, gaze-based toggles and on-screen rating and correction tools—that allowed the system to adapt to the participant’s needs over time, including his shift from vocalized to silent speech. Finally, we automated the system startup and shutdown so that trained care partners could don and doff the system with a few simple steps. We also optimized the system so that, once running, it could operate continuously for up to 19 h without any care partner intervention.

The participant used this multimodal BCI system as his preferred mode of communication and digital access, instead of existing options such as a gyroscopic head mouse or expertly-interpreted translation through a care partner. He used this system in a variety of personal and professional contexts; the richness and duration of use in this study provide strong evidence that intracortical BCIs can offer not just high peak performance in research settings but also stable, versatile support for communication and digital interaction during independent home use.

This study has several limitations. It involved a single participant, and the generalizability of these results to other individuals, electrode implant sites, intracortical electrode types or neurological conditions is not yet known. The system relied on percutaneous wired connections and required daily setup by trained care partners, which may limit broader adoption. The multicomputer system’s bulkiness also limited portability and prevented use outside of the participant’s home. We did not systematically assess user fatigue or long-term device wear, although the system’s continued high decoding performance suggests durability across the timescales studied. Future work will be needed to evaluate wireless or fully implantable systems, minimize setup time and expand access to users with different clinical profiles. Finally, speech decoding accuracy achieved during independent conversation was not consistently as high as it was during structured research sessions with prompted sentences. Work remains to deliver a speech neuroprosthesis with the same consistency and accuracy as healthy speech across the full range of what the user is trying to say.

Ultimately, this study demonstrates that stable, high-performance intracortical BCIs can be used independently and productively in a real-world setting, reliably supporting both communication and digital access over nearly 2 years of use. These findings mark a substantial step toward practical, long-term assistive neurotechnologies for individuals with severe motor and speech impairments.

## Methods

### Clinical trial and participant

This study includes data from a single participant (‘T15’) enrolled in the BrainGate2 clinical trial (NCT00912041). This paper does not report primary clinical-trial outcomes; instead, it describes scientific discoveries that were made using the data collected in the context of the ongoing clinical trial. BrainGate2 is a sponsor–investigator-led multicenter, open-label, FDA-approved IDE study (IDE no. G090003) assessing the safety of chronically implanted intracortical electrodes (‘Utah’ arrays) in individuals with paralysis. The trial was approved by the institutional review boards at Mass General Brigham (protocol no. 2009P000505) and the University of California, Davis (protocol no. 1843264). T15 provided written informed consent to participate in the trial and to publish photographs and video recordings. All procedures were conducted in accordance with relevant guidelines and regulations.

T15 is a man with ALS who was 45 years old at the time of his enrollment in 2023. He retained eye and limited neck movements but was severely dysarthric. His existing means of communication included communicating through expert interpreters (6.8 WPM) or typing words out letter-by-letter using a gyroscopic head mouse (Quha Zono 2, 6.3 WPM). Further details of T15’s clinical status were previously reported^5^.

In summer 2023, four 64-electrode silicon microelectrode arrays (Utah arrays; 1.5-mm tine length, iridium-oxide coated; Blackrock Neurotech) were placed in T15’s left precentral gyrus targeting speech motor cortex. Specific information regarding how implant locations were chosen was previously described^5^. Arrays were connected via subcutaneous wires to a percutaneous titanium pedestal affixed to the skull.

### Neural signal recording, processing and feature extraction

Neural activity was recorded from 256 electrodes using Neuroplex E headstages (Blackrock Neurotech), connected to the two percutaneous pedestals (each linked to two implanted microelectrode arrays). Signals were analog-filtered between 0.3 Hz and 7.5 kHz (fourth-order Butterworth), digitized at 30 kHz with 250-nV resolution and streamed in 1-ms windows to a custom real-time processing script written in Python version 3.8. Each 1-ms window was band-pass filtered between 250 and 5,000 Hz using a fourth-order zero-phase Butterworth filter. To reduce edge artifacts, windows were padded using the previous 1 ms of data on the left and mean-padding on the right. Linear regression referencing^28^ was applied independently to each array (64 electrodes per group) to suppress common-mode noise.

Two standard neural features were extracted from each electrode: threshold crossing spikes and spike-band power. Threshold crossings were identified when the voltage crossed −4.5 × the root mean square (r.m.s.) value for that electrode. Spike-band power was computed by squaring the filtered signal and averaging over the 1-ms window, with a cap of 12,500 μV^2^ to reject outliers. This full preprocessing pipeline—including filtering, denoising and feature extraction—was completed in under 1 ms per window. Extracted features were binned into 10–20-ms nonoverlapping intervals, depending on the input requirements of each decoder. Binned threshold crossings were computed by summing across consecutive windows; binned spike-band power was calculated by averaging over the same span.

At the beginning of each session, a brief calibration task involving repeated word attempts was used to estimate per-electrode r.m.s. values for thresholding and to compute linear regression referencing filter coefficients. These parameters were re-estimated after each block of recording throughout the session to mitigate signal nonstationarities over time.

### Data collection rig

The BCI system was implemented using a distributed, multicomputer setup designed to support both high-bandwidth neural data collection and low-latency, real-time multimodal neural decoding and user interfaces^22^. Although the current configuration includes more computational power and physical footprint than would be needed in a future system suitable for widespread clinical use, it enabled rapid prototyping, flexible experimentation and continuous decoder optimization. Although this large wheeled computer rack system limits portability, T15 was able to use it throughout his home. In future iterations, the system could be miniaturized into an embedded platform with a portable external interface, similar to prior efforts in reach-and-grasp BCIs using Utah arrays^14^.

Real-time data acquisition and decoding were managed by four networked computers. A Windows 10 machine interfaced with the Neuroplex E headstages to control neural recording. A second computer (running Ubuntu 22.04 LTS) processed raw 30-kHz neural data, extracted features and streamed them to a third machine (Ubuntu 22.04 LTS) responsible for GPU-intensive operations including real-time decoder inference, online decoder fine-tuning and user-facing task display. A fourth computer (Ubuntu 22.04 LTS) ran the phoneme-to-word language models that generated final text output. All machines were connected via a local area network and synchronized using the Backend for Real-Time Asynchronous Neural Decoding (BRAND) framework^22^. The code was implemented in Python, C and MATLAB.

### Eye tracking

T15’s eye gaze was tracked using a Tobii Pro Spark eye tracker (Tobii AB) that was mounted at the bottom of the BCI system participant monitor. Calibration was performed at the start of each session (for both research sessions and independent sessions) and repeated as needed via an option available in the BCI system menu (Supplementary Video 13). Gaze control typically required one calibration routine at the beginning of each day, plus potential additional recalibrations if T15 moved, if the monitor was moved or if lighting conditions changed. The gaze calibration routine took less than 30 s and could be accessed at any time through the BCI user interface’s menu screen. Gaze location was sampled at 60 Hz, averaged across both eyes, smoothed over time and used to enable gaze-based selection of on-screen buttons. Button activation was triggered by maintaining gaze on an on-screen target for 0.5–1.0 s.

To compare T15’s neural cursor control with gaze-based control, we asked him to perform the same grid task (14 × 14 grid where each tile measures 0.77 inches × 0.77 inches on screen) using gaze-based control. His bit rate was 0.80 ± 0.33 bits per second. With larger grid tiles (6 × 6 grid, 1.79 inches × 1.79 inches per tile), T15 made selections at 1.67 ± 0.21 bits per second with neural cursor and 2.59 ± 0.26 bits per second with gaze control.

To improve usability and reduce selection errors, we implemented a custom ‘magnetization’ feature that subtly attracted the gaze cursor to the center of nearby on-screen buttons, making them easier to select. Eye tracker calibration, gaze data acquisition and selection logic were implemented in custom Python software integrated into the BRAND-based data collection platform.

### Data collection sessions

All data for this study were collected in one of two types of data collection sessions: scheduled research sessions or personal use sessions. All data collection sessions occurred in the participant’s home. Scheduled research sessions occurred 0–2 times per week, and during these sessions, the participant would be asked to do a research-focused task that, for the purposes of this study, most often involved attempting to say prompted sentences aloud in a structured copy task. By contrast, personal use sessions had no research-focused tasks other than what was necessary for enabling use of the BCI for the participant’s self-directed communication and digital access. Before post-implant day 281, personal use sessions were scheduled 0–4 times per week and required a member of the research team (co-author C.I.) to be present to don and doff the system. After post-implant day 281, following an amendment to the IDE, T15 and his care partners could use the BCI system whenever they wanted to without researcher assistance. Data from all sessions, except for when ‘privacy mode’ was enabled during personal use sessions, were saved and used to train future speech decoding models. This study includes data from post-implant days 25–678. Supplementary Videos 4–13 were recorded on a more recent day (post-implant day 874), which was included as a filming session to demonstrate key features of the BCI system.

### Speech decoding

#### Neural feature preprocessing

Neural activity was decoded into text using two types of neural network architectures used over the course of the study: an RNN and a transformer-based model. Both architectures processed 512-dimensional neural feature vectors (threshold crossings and spike-band power from 256 electrodes, binned every 20 ms), which were *z*-scored using statistics from the preceding 5–20 speech trials and smoothed using an acausal Gaussian kernel (*σ* = 40 ms), where *σ* is the smoothing strength parameter of a Gaussian filter.

#### Phoneme classification

Both models were trained to predict a distribution over 41 output classes: the 39 standard American English phonemes plus a ‘silence’ class and a connectionist temporal classification (CTC) blank token. This formulation enabled alignment-free training using the CTC loss function^7^. Sentence labels for each trial were converted to phonemes using the CMU pronunciation dictionary^29^ and the *g2p-en* Python package^30^.

#### RNN decoder architecture and pretraining

A RNN predicted phoneme probabilities from neural activity. The model consisted of three components: (1) a day-specific linear input layer to account for intersession neural drift, (2) five stacked GRU layers, each with 768 units, and (3) a dense output layer producing a distribution over 41 classes. The RNN produced outputs every 80 ms on the basis of the most recent 280 ms (14 bins) of neural data.

Offline model pretraining was performed before each session using all previously collected data. Training used 90% of past trials and randomly held out 10% per day for validation. Each batch (up to 64 trials) was sampled from one session, with real-time data augmentation including white noise and constant offsets. The optimization was done with Adam (learning rate linearly decayed from 0.02, *β*_1 _= 0.9, *β*_2 _= 0.999 and *ε* = 0.1), with dropout and L2 regularization.

#### Transformer decoder architecture and pretraining

On post-implant day 600, we switched from using the RNN-based decoder to a more accurate transformer-based decoder. We did not perform a formal multiple-repetitions evaluation of the effect of switching decoder architectures. However, we did not observe any temporary reduction in decoding performance (Fig. 2a,e), which is consistent with the quasi-open loop nature of the brain-to-text BCI.

The transformer-based decoder consisted of three stages: day-specific transformation, input striding and a 12-block causal transformer with multihead attention. Day-specific embedding input layers added a learned day-specific vector to each input timestep to correct for interday variability. This linear offset (as opposed to a nonlinear transformation as was used for the RNN^5,7^) was empirically found to perform better for this architecture. The transformer decoder applied multiheaded causal attention with rotary positional embeddings^31^ and used RMSNorm^32^ for normalizing hidden states. Outputs from the transformer were fed through a feedforward layer to produce phoneme probability distributions every 80 ms. Similar to the RNN, this model was also trained with CTC loss, which automatically aligned neural data and labeled phoneme sequences.

Offline model pretraining used all previously recorded labeled data (that is, copy task trials (Supplementary Video 4) and personal use trials that were confirmed by the participant to be decoded correctly), excluding trials that had excessive electrical artifacts or were too long, with a 90–10 train–validation split per day. Data were augmented with noise and constant offsets, and then smoothed using a Gaussian kernel (*σ* = 40 ms). Mixed-day batches (six random days of data, 16 randomly-selected trials from each) were used to regularize learning. The optimization was performed with AdamW using a cosine decay schedule and warmup. The offline model pretraining lasted for 24–36 h (400,000–600,000) batches and was run on a single RTX 4090 GPU.

#### Continuous fine-tuning (both models)

During real-time use, both RNN and transformer models underwent continuous fine-tuning. Once a minimum number of new sentences were collected (5–6), a new day-specific embedding was created. Each new sentence had a 67% chance of entering the training buffer and 33% chance of being held out for validation. Fine-tuning batches (size 64) drew data from the current and three random prior days. Checkpoints were updated only if validation performance improved. Fine-tuning occurred in 10-batch intervals for up to 500 batches per session using cosine learning rate decay. This continual adaptation improved the decoding stability and accuracy by tracking nonstationarities across sessions.

#### Speech decoding latency

**Extended Data Fig. 8:**
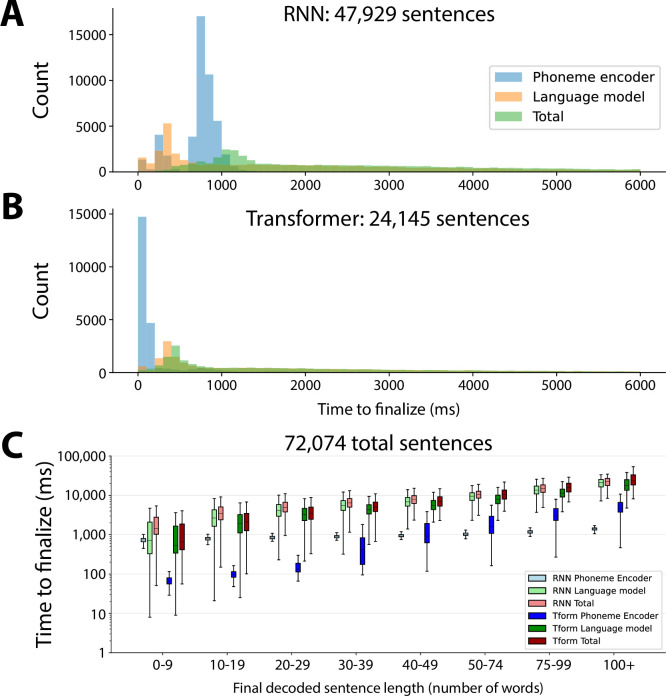
Sentence finalization latency. (**a**) Distribution of sentence finalization latencies across 47,929 personal use sentences using the RNN-based phoneme encoder. (**b**) Distribution of sentence finalization latencies across 24,145 personal use sentences using the transformer-based phoneme encoder. (**c**) Sentence finalization latencies presented as a function of the number of words in the final decoded sentence (standard box and whisker plots).

Decoding latency can be separated into two categories: real-time feedback during attempted speech and sentence finalization after the participant finished speaking. During attempted speech, latency was primarily attributable to acausal Gaussian smoothing (160 ms) and the input patching strategy (80 ms), for an estimated total of 160–240 ms during real-time inference. Real-time phoneme decoder and language model inference each typically took less than 1 ms. Sentence finalization involved renormalizing and resmoothing neural features, rerunning phoneme decoding, beam search decoding with an unpruned n-gram model and large language model rescoring of candidate word sequences. The RNN-based phoneme encoder had a median finalization latency of 2,657 ms (772-ms phoneme encoding, 1,921-ms language model; Extended Data Fig. 8a), whereas the transformer-based encoder had a median finalization latency of 1,791 ms (85-ms phoneme encoding, 1,623-ms language model; Extended Data Fig. 8b). Finalization latency scaled with sentence length: sentences under 20 words took 2,072 or 1,171 median milliseconds (RNN and transformer, respectively), whereas sentences of 20 words or longer took 6,258 or 5,181 median milliseconds (Extended Data Fig. 8c).

### Cursor decoding

#### Imagery

During neural cursor control, we initially instructed T15 to attempt to move his right arm and hand as if he were controlling a computer mouse, although he noted that, over time, he stopped thinking about moving and instead relied on ‘intuition’ to simply move the cursor^17^.

#### Calibration task

The cursor calibration task could be initiated at any time through the user interface menu (Extended Data Fig. 5 and Supplementary Videos 3 and 10). Cursor decoders were only updated during this calibration task and did not undergo background fine-tuning. Until post-implant day 621, the calibration task was a center-out-and-back eight target ‘radial 8’ acquisition task with fixed target distance (40% of screen height), fixed target radius (5% of screen height) and the next target predictably in the center after every outward trial^17^. After day 621, the task was made more varied and engaging to elicit more diverse cursor movements (similar to the previously reported Fitts task^33^). Target positions could be anywhere in the two-dimensional (2D) workspace (1,920 pixels × 1,080 pixels), leading to both shorter and longer target distances, and cursor trajectories at any angle. Target radii were smaller than in the radial 8 task and varied (2.5–4.0% of screen height).

#### Decoder architecture and training

Cursor control originally used a linear velocity decoder as reported previously^18^, which T15 calibrated anew each day. After post-implant day 467, the decoder’s speed gain and nonlinear speed adjustment cutoff were set to new values, which improved T15’s ability to stop on and click small buttons during computer use. This improvement was reflected in a high-performance grid evaluation task, which inherently rewards precision^9,18,33–37^ but was not reflected in the performance on the calibration task (Fig. 3e), whose larger targets tolerated faster cursor movements at the cost of precision.

After day 654, cursor control used an RNN-based decoder. The RNN was trained offline on up to 44 prior sessions of cursor calibration data. Unlike the linear decoder, the RNN was not updated online during calibration. However, the calibration task was still used daily to train a click decoder and to collect up-to-date input feature statistics (spike band power means and standard deviations) for input normalization.

Each input to the RNN was a 256-dimensional vector of *z*-scored spike band power for a 10-ms time bin. Every 10 ms, the decoder output a 2D vector $\{{\bf{x}},{\bf{y}}\}$ representing a predicted cursor velocity, and then moved T15’s cursor accordingly. The RNN model consisted of a two-layer GRU with a hidden size of 64, followed by a dense layer projecting the hidden state to a 2D output vector (Extended Data Fig. 3).

During training, labels were 2D vectors from cursor to target (‘target vector’). The target vector was set to $\{{\bf{0}},{\bf{0}}\}$ when the cursor was touching the target under the assumption that the participant was trying to hold still^38,39^. Training aimed to minimize the per-sample loss function

$$\ell =|{v}_{\mathrm{perpendicular}}|-\,\max (|{v}_{\mathrm{parallel}}|,1),$$

where ${v}_{\mathrm{perpendicular}}$ is the component of the predicted velocity $v$ that was perpendicular to the target vector, and ${v}_{\mathrm{parallel}}$ is the component of the predicted velocity $v$ that was parallel to the target vector. This loss function encouraged output vectors that pointed toward the target, with a bounded magnitude. This loss function also encouraged output vectors of $\{{\bf{0}},{\bf{0}}\}$ while the cursor was touching the target. Each training epoch, Gaussian noise was added to both the inputs (*z*-scored spike band power) and labels (target vectors), as a form of data augmentation^26^.

**Extended Data Fig. 9:**
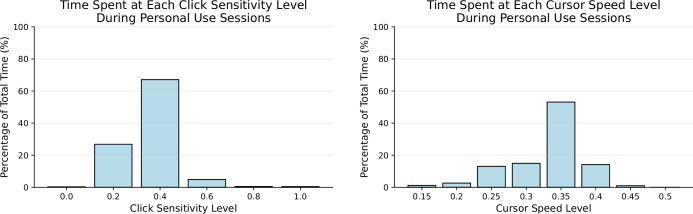
Neural click sensitivity and neural cursor speed settings. T15 could adjust the neural click sensitivity and the neural cursor speed within the BCI user interface. Data shown is from 126 days (1208.2 h) of personal use wherein T15 used the neural cursor and the click sensitivity and cursor speed metrics were tracked. Left: Distribution of click sensitivity values. Click sensitivity initiated to 0.20 when the BCI system was initialized, and could be adjusted up and down in increments of 0.20 (bounded between 0 and 1). Across all data, the average click sensitivity was set to 0.36 ± 0.12 (mean ± standard deviation). Right: Distribution of cursor speed values. Cursor speed initiated to 0.40 when the BCI system was initialized, and could be adjusted up and down in increments of 0.05 (bounded between 0 and 1). Across all data, the average cursor speed was set to 0.33 ± 0.05.

During online cursor control, output vectors were scaled by a speed gain *β*, which T15 could raise or lower using the on-screen user interface (Extended Data Figs. 5h and 9, right).

### Click decoding

Click decoding used a linear decoder as reported previously^17^, which T15 calibrated anew each day as part of the same tasks used to calibrate cursor control. T15 was initially instructed to attempt to squeeze his right fist for a click, although as with cursor control, he reported transitioning to a more intuitive strategy over time^18^. The decoder’s weights were computed online using logistic regression every 3.0 s during calibration.

Each input to the click decoder was a 512-dimensional vector of *z*-scored neural features (256 threshold crossings values and 256 spike band power values) for a 10-ms time bin. Every 10 ms, the decoder output a discrete class ‘click’ or ‘no click’, and the BCI performed a left-click on T15’s computer for each ‘click’ decoded.

To reduce false positive click detections, a ‘click’ was only decoded when the linear model’s probability estimate for ‘click’ was higher than for ‘no click’ for some minimum portion $T$ of time bins within a 70-ms window. T15 could raise or lower the click sensitivity ($1-T$) using the on-screen interface (Extended Data Figs. 5h and 9, left).

### Decoder evaluations

#### Speech decoding

Consistent with prior studies^5,7,10,12,40^, we benchmarked our speech decoding accuracy by calculating the predicted phoneme and word error rates during a structured copy task where the participant was asked to say prompted sentences aloud, thereby allowing us to directly compare the decoded output with the ground truth target sentence. Word and phoneme error rates were calculated using Levenshtein distance, which counts the number insertions, deletions or substitutions necessary to match the decoded phonemes or words to the ground truth labels. Reported error rates were aggregated across all evaluation sentences from each session by summing the number of errors (insertions, deletions or substitutions) for all sentences and then dividing it by the total number of words in those sentences. This helps prevent very short sentences from overly influencing the result. Confidence intervals for error rates were computed via bootstrap resampling over individual trials and then recalculating the aggregate error rates over the resampled distribution (10,000 resamples).

During independent BCI use, ground truth was unavailable; therefore, T15 used gaze or cursor control to label each decoded utterance as ‘100% correct’, ‘one word wrong’, ‘mostly correct’ or ‘incorrect’, with the option to make corrections before rating. He was informed that these labels would be used for ongoing recalibration and system improvement, incentivizing accurate reporting. T15 told us that he pressed the wrong accuracy rating button by accident on less than 1% of sentences, although we note that we did not have a way to independently check the ground truth of independent use sentences. T15 reported using a threshold of approximately two thirds of words decoded correctly to distinguish ‘mostly correct’ from ‘incorrect’ (Supplementary Table 1). Examples of sentences with each rating are in Table 1.

#### Cursor decoding

Formal evaluations of cursor decoding performance were done in structured research sessions via a grid task^9,18,34–37^ from which we could compute bits per second. During daily independent BCI use, however, the only structured cursor-related tasks that the participant performed were the center-out-and-back or random target acquisition tasks, which were used to calibrate the cursor decoder. Thus, we relied on metrics from these tasks to quantify the daily cursor decoding performance. These measures included (1) how long it took the participant to achieve full closed-loop cursor control during the calibration task, (2) the target acquisition rate during the calibration task and (3) the total minutes of calibration per day.

### BCI system software (user interface)

We built a custom user interface that supported the daily independent use of the BCI for speech decoding, cursor control, personal computer control and a variety of related options. The user interface was primarily coded with the Pyglet Python package version 2.0.1240 and consisted of a range of function-specific ‘pages’ that the user could navigate between using gaze or cursor control (Extended Data Fig. 5). This interface was displayed on a monitor that was mounted on an articulating arm, which was positioned above the participant’s personal computer (for example, Fig. 3a). The system was periodically updated in response to participant feedback, feature requests and bug fixes in the underlying custom software stack (Supplementary Table 2). Additional details about the user interface and T15’s feedback on specific design features is available at ref. ^41^.

While idle, the system waited to detect attempted speech or a user interface button press via gaze or cursor. During attempted speech, predicted words were displayed on screen in real time. After speaking, the participant could make corrections to the decoded word sequence, rate its accuracy and optionally have it played as audio via text-to-speech or typed into the active text field on his personal computer. A menu screen provided access to additional features including cursor and eye tracker calibration, cursor speed and click sensitivity adjustment, a full gaze- or cursor-controlled keyboard, a toggleable profanity filter and a privacy mode that prevented data logging. Full details of the user interface are described in ref. ^41^ and shown in Extended Data Fig. 5.

### BG Home software (personal computer integration)

A custom application (‘BG Home’) enabled our BCI system to control the participant’s computer’s mouse and key presses such that he could use his personal computer via BCI control. It consisted of a small user feedback window hovering in the corner of the computer screen, plus an asynchronous background process which received streaming data from the BCI system and performed the mouse movement and key presses (Supplementary Video 12). BG Home was built with Electron but used Python for the background process. A snapshot of what the BG Home software looks like while the participant is attempting to speak can be seen in Fig. 3a.

### Statistical analyses

Results for each analysis are reported as means and either 95% confidence intervals or standard deviations. Confidence intervals were estimated by randomly resampling each dataset 10,000 times with replacement and have not been adjusted for multiplicity. The measures used for the evaluation of speech decoding performance (phoneme error rate and word error rate) were chosen before the start of data collection. For statistical comparisons, parametric methods were used if the relevant assumptions were satisfied; otherwise, nonparametric tests were used.

### Reporting summary

Further information on research design is available in the Nature Portfolio Reporting Summary linked to this article.

## Online content

Any methods, additional references, Nature Portfolio reporting summaries, source data, extended data, supplementary information, acknowledgements, peer review information; details of author contributions and competing interests; and statements of data and code availability are available at 10.1038/s41591-026-04414-6.

## Supplementary information

Supplementary Information Supplementary Tables 1 and 2.

Reporting summary

Peer Review File

Supplementary Video 1 T15 describes the impact of the BCI on his life. T15 uses the speech BCI to describe how the system impacts his everyday life. He is navigating the user interface with eye gaze, which is rendered on screen as a semi-transparent white circle. His attempted speech is automatically detected by the speech decoder, and the decoded words are displayed on screen in real time. The BCI system monitor is on an articulating monitor arm, which is mounted on his desk and positioned above his personal computer screens. This video was recorded on post-implant day 678.

Supplementary Video 2 T15 converses with a researcher. T15 uses the speech BCI to talk to a researcher (author N.S.C.), making jokes about the speech decoding system. He is navigating the user interface with eye gaze, which is rendered on screen as a semi-transparent white circle. At the end of each sentence, he rates the accuracy of the decoded text and has an opportunity to make corrections if necessary. He also uses the text-to-speech functionality to read the decoded words aloud in a digital voice that was optimized to sound similar to his pre-ALS voice. This video was recorded on post-implant day 606.

Supplementary Video 3 T15 calibrates the cursor and click decoders. T15 uses eye gaze to navigate the BCI system’s user interface, going from the main speech interface, to the main menu, to a cursor calibration submenu and, finally, to the cursor calibration screen. Using the RNN-based cursor decoder, T15 then does the calibration task, wherein he attempts to move the cursor (white circle) to the target (green circle). When the cursor arrives at the target, T15 selects the target with either a dwell or a click, as instructed by the task. Throughout the calibration task, the cursor and click decoders are constantly updated, and the angle error of the decoded cursor movement direction is displayed on screen. When T15 is satisfied with the cursor calibration, he can exit this screen using gaze control by simply looking at one of the corners of the screen. This video was recorded on post-implant day 649.

Supplementary Video 4 T15 calibrates the speech decoder. T15 navigates the user interface (using eye gaze) to enter the speech calibration routine, where he attempts to say prompted sentences. Neural data during these prompted sentences are then used to fine-tune the speech decoder in the background. This video was recorded on post-implant day 874.

Supplementary Video 5 T15 converses with a researcher. T15 jokes around with a researcher (author N.S.C.). T15 is using eye gaze to navigate the user interface, and he rates the accuracy of each decoded sentence after it is finalized. Neural data from correctly decoded sentences are used to fine-tune the speech decoder in the background. This video was recorded on post-implant day 874.

Supplementary Video 6 Spelling-based speech decoding. T15 demonstrates spelling mode, where he can attempt to say sequences of letters instead of words to spell out a word. T15 is using eye gaze to navigate the user interface, and he rates the accuracy of each decoded letter sequence after it is finalized. Neural data from correctly decoded letter sequences are used to fine-tune the speech decoder in the background. This video was recorded on post-implant day 874.

Supplementary Video 7 Privacy mode. T15 demonstrates privacy mode. When privacy mode is enabled, it is denoted by a red border around the screen. T15 can still use the system as normal in privacy mode, but no data (for example, neural features, decoded sentences and so on) will be saved. T15 can enable or disable privacy mode at any time. In this video, T15 is using eye gaze to navigate the user interface. This video was recorded on post-implant day 874.

Supplementary Video 8 Sentence history. T15 demonstrates the sentence history screen, where he can select from recently decoded sentences to retype them onto his personal computer or have them be read aloud as text-to-speech (post-implant day 874). In this video, T15 is using eye gaze to navigate the user interface. This video was recorded on post-implant day 874.

Supplementary Video 9 Sound effects. T15 demonstrates sound effects, which is a feature that he requested and is customized to him. When certain key words are decoded by the speech decoder and he plays them aloud with text-to-speech, corresponding sound effects are played aloud. For example, ‘sad trombone’ will play a sad trombone sound. The sound effect feature can be enabled or disabled via a toggle button in the menu. In this video, T15 is using eye gaze to navigate the user interface. This video was recorded on post-implant day 874.

Supplementary Video 10 Neural cursor calibration. T15 does the neural cursor calibration task, wherein he attempts to move the cursor (white circle) to the target (green circle). When the cursor arrives at the target, T15 selects the target with either a dwell or a click, as instructed by the task. The task begins with cursor and click assistance enabled, but these scale down as T15’s cursor control improves. Throughout the calibration task, the cursor and click decoders are constantly updated, and the angle error of the decoded cursor movement direction is displayed on screen. When T15 is satisfied with the cursor calibration, he can exit this screen using gaze control by simply looking at one of the corners of the screen. In this video, T15 is using eye gaze to navigate the user interface. This video was recorded on post-implant day 874.

Supplementary Video 11 Navigating the BCI user interface with neural cursor control. T15 first uses eye gaze to enable neural cursor control. When neural cursor control is enabled, a mouse cursor is rendered on the screen, and when T15 attempts to click, a growing blue circle appears at the tip of the cursor. Note that, even when neural cursor control is enabled, T15’s eye gaze is still rendered on the screen as a semi-transparent white circle, although it is not used for actually navigating the user interface. This video was recorded on post-implant day 874.

Supplementary Video 12 Personal computer control via BCI. T15 demonstrates using neural cursor and speech decoding to browse the web on his personal computer. T15 uses eye gaze on the BCI system user interface to enable ‘PC cursor’ and move and click the mouse on his personal computer. He also uses eye gaze to copy and paste recently decoded sentences from the BCI system to his personal computer. This enables him to open a web browser, navigate to Wikipedia and search for BCI-related articles. Outside of this video, he uses the same strategy to perform a wide range of functions on his personal computer. This video was recorded on post-implant day 874.

Supplementary Video 13 Eye tracker calibration. T15 selects the eye tracker calibration routine from within the BCI user interface menu and then performs the calibration. He looks at the red circles as they appear, and then a results screen is briefly shown. In this video, T15 is using eye gaze to navigate the user interface. If eye gaze tracking were not accurate enough for T15 to navigate to the eye tracker calibration routine in the first place, a care partner could assist him using a computer mouse. This video was recorded on post-implant day 874.

## Data Availability

Neural data and speech decoding transcripts recorded during the participant’s independent use of the BCI system cannot be shared to preserve the participant’s privacy. Data to reproduce the figures and statistical results in this manuscript are available via GitHub at https://github.com/Neuroprosthetics-Lab/NatMed_independent_personal_use.

## Code availability

Code to reproduce the figures and statistical results in this manuscript are available via GitHub at https://github.com/Neuroprosthetics-Lab/NatMed_independent_personal_use.
