# Supplementary material for: Long-term independent use of an intracortical brain–computer interface for speech and cursor control
Source: Nat Med. 2026 Jun 15;32(7):2504–10. doi: 10.1038/s41591-026-04414-6 (PMC13375540; doi:10.1038/s41591-026-04414-6)
Supplement: Supplementary file 1 — Supplementary Tables 1 and 2. [file 41591_2026_4414_MOESM1_ESM.pdf]

nature medicine

Supplementary information https://doi.org/10.1038/s41591-026-04414-6

Long-term independent use of an intracortical brain–computer interface for speech and cursor control

In the format provided by the authors and unedited

Table S1. Questions and answers with T15.

Question	T15's answer
Please briefly explain your rationale for how and when you rate the accuracy of decoded sentences. The "100% correct" and "one word wrong" ratings are self-explanatory, but how do you decide between rating a sentence "mostly correct" or "incorrect"? For example, a 10-word sentence that has 2 words wrong might be "mostly correct", but a 3-word sentence that has 2 words wrong might be "incorrect".	I use a percentage of two thirds to assess whether something is good enough to be mostly correct. It needs to be that or higher.
How frequently do you turn off the sentence rating feature, and what are the typical reasons for doing so (general reasons are fine, I don't need specifics)?	Probably about three or four times a week, and always for something connected to physical comfort. I may be moving my chair too far away to have access to the eye tracker technology, so I will do this for simplicity. It will save me time, or alternatively, I will want to be able to talk very quickly for emergencies. I will turn it off for this reason. But likely less than one percent of my overall sentences and such.
When a sentence is not decoded completely correctly, how do you decide whether to enter the correction interface or to skip it and mark the sentence as "one word wrong", "mostly correct", or "incorrect"?	I would normally check whether a one mistake sentence is able to get changed into a one hundred percent correct sentence. And after you told me how important this is, I have been checking every time. On the other hand, if the sentence is completely hot garbage, I will label it incorrect before I check it. The longer the sentence, the more likely it will be eighty five percent or higher correct. Most sentences that are more than ten words and are labeled mostly correct are in between eighty five to ninety five percent correct. FYI I am usually only skipping the sentences that are obviously missing the right count of phonemes. The decoder will get off track and the sentence will be completely ruined.
When you choose to enter the correction screen, roughly what percentage of the time do you think you are able to successfully correct the sentence?	I think I know what you are looking for here. I am always almost able to correct a word or two in a sentence with multiple mistakes. But I think that I am able to get a sentence all the way to one hundred percent correct about thirty to forty percent of the time.
Since we implemented the upgraded brain-to-text decoder [on post-implant day 602], have you noticed improved or more reliable decoding accuracy?	Yes, it is difficult to give quantitative feedback about this, but the answer is a resounding yes. I would say the amount of sentences that are one hundred percent correct has improved, as well as the number of hard to decode words being correctly decoded.
Do you notice any trends between decoding accuracy and (a) how you are feeling or (b) what you are trying to talk about?	I think that it is a relatively narrow and very high range of different days I experience in terms of decoding accuracy. So, while the answer is yes, it is not something that I am saving a particular conversation for when the decoder is

	having a good day. Being able to fully communicate to people is a dream. We are not there yet, but this is such a massive step forward and every day that we can improve something, even a little tiny bit is felt by me as something really positive and will impact my mood and my day, absolutely.
How has your overall experience with personal use been in the last few months? How does it compare to last year?	It feels like we are still on the upward swing. With a dedicated team of people working on this, we are still making improvements on the utility of this service. Every day is a new challenge to solve, but I feel like we are only getting better. This would be very different if I was going down hill and losing my ability to talk.
Broadly, what do you typically use our system for throughout a day, in terms of controlling your own personal computer? For example, could you estimate the number of text messages (texts, slack, emails) you might send in a day, or whether you use it for video calls, or just to relax and watch a show or search the web?	I will send a lot of very short messages on multiple points during the day. Probably twenty to thirty one line messages. slack. signal. text. email. Then I would probably have two or three longer messages, like this email that I will send in a day. Or I will make a similar contribution on a Google document or sheet. Definitely will get in a video or two as a little treat. I will use the device to tell [my assistant] what to do and give him feedback. During the work week, we will definitely hop on a few calls every day. And on the weekends or the evening, I will have visits from friends and family, or usually I will call with them. I will use the internet to get everything because I do not usually leave my house. So that is incredibly important to be able to stay on top of my finances and shopping. It will take me more time, but I am basically using my computer in the same way as I was doing before the disease.
Any other comments you want to share that my questions did not cover?	Look at the very small or short sentences, about three words or less, and you know that the decoder will work much better with more context. And I would think that the majority of completely incorrect sentences are coming from my obsession about trying to get the exact right word. If you take those sentences out, you will get very different data.

Table S2. Timeline of updates and changes to the BCI system.

Post-implant day	Feature	Description
147	Bluetooth keyboard	Enables the BCI system to act as a bluetooth keyboard for the participant's personal computer so that they can type decoded text into text fields on their computer.
227	Sentence correction	Enables the user to choose between the top 5 most likely decoded sentences if the top sentence was not decoded correctly.
232	Eye tracker button magnetization	"Magnetizes" on-screen eye tracker buttons so that tracked gaze will gravitate toward them when nearby. This makes gaze-based control easier to use and more robust to imperfect calibration.
281	Independent mode	The system was automated so that care partners could power it on and off without researcher assistance.
295	BG Home version 1	Version 1 of the BG Home software suite was deployed, which integrates the BCI system directly with the user's computer.
303	Added "one word wrong" sentence rating option	At the participant's request, we added the "one word wrong" sentence rating option alongside the existing "100% correct", "mostly correct", and "incorrect" options.
358	Enabled cursor control for personal computer	This update involved adding a new menu screen to the BCI system UI, a cursor calibration game, and a major update to the BG Home software that supported controlling mouse inputs.
361	Use recent context to help predict the current decoded sentence.	Recently decoded sentences are fed as context into the language model alongside the current most likely candidate sentences to help choose the most contextually appropriate option. Recent context is also displayed on the screen to help conversation partners keep up with the current topic of conversation.
364	Eye tracker calibration	Enabled the participant to initiate an eye tracker calibration game via the gaze- or cursor-based user interface without care partner assistance.
412	Speech decoder background calibration bug fix	A major bug was identified and fixed in the background calibration code for the speech decoder, which resulted in immediately increased speech decoding accuracy during personal use.
462	Word-based correction	Instead of making corrections by choosing among the top 5 most likely decoded sentences, the participant can now choose individual words that were decoded incorrectly and replace them by choosing from a list of contextually appropriate replacement words.
462	Privacy mode	The participant can enable a privacy mode via the gaze- or cursor-based interface. When enabled, no data will be saved.
467	Optimized cursor parameters	The cursor decoder's speed parameters were set to more optimal values for better precision (selecting tiny buttons) and traversal (moving across the screen).
484	Miscellaneous updates	Visual user interface refinements, a bad-word language filter, keyword sound effects (Video 9), ability to disable speech detection, ability to disable sentence rating.
486	Optional cursor control on BCI system user interface	Instead of relying on the eye tracker exclusively, the participant can now also use cursor control to navigate the BCI system user interface.
491	Refresh word suggestions	Enabled the participant to generate new updated word suggestions during word correction mode. This is useful for when he makes some corrections, then can refresh to get more contextually relevant options

		before continuing to make additional corrections.
505	Word insertion and deletion	Enables the participant to insert or delete words from the decoded sentence during word correction.
561	Speech detection animation	Added a user interface element that animates (grows and shrinks in size) whenever speech is detected by the speech decoder.
563	Care partner UI assistance	Added the ability for a care partner to use a physical mouse to navigate the BCI system user interface in the event that the participant needs help (e.g., if gaze control stopped working and needs to be recalibrated).
565	Gaze- or cursor-based full keyboard	Added a full keyboard that can be controlled with gaze or cursor so that the participant can type things on his personal computer that would be difficult or impossible to do through the speech decoder (e.g., a website address).
565	Automatically pause computer cursor control during gaze	When the participant's gaze is detected on the BCI system screen, computer cursor control is paused so that errant cursor movements or clicks do not occur.
588	Aesthetic user interface changes	Font was changed throughout the system. The gaze selection circle on the word correction screen was animated to make it more clear when a dwell selection was complete.
602	New transformer-based brain-to-text decoder	A transformer-based brain-to-text decoder was deployed, resulting in higher speech decoding accuracy with less calibration required.
612	Cursor- or click- only calibration modes.	The participant can now choose whether to calibrate the cursor decoder, click decoder, or both. Prior to this change, both decoders always had to be calibrated in tandem.
616	Updated word correction screen UI	Updated and optimized the user interface for the word correction screen to provide a more seamless user experience.
621	New cursor calibration game	Switched from a center-out-and-back style cursor calibration game to a varied target task to get more diverse cursor training data.
624	More word correction options	During word correction, the participant is now offered a wider variety of potential replacement words.
651	Refactored user interface code	Refactored the entirety of the BCI system user interface code, resulting in higher frame rates and a variety of bug fixes.
654	Upgraded to RNN-based cursor decoder	Upgraded the cursor decoder from a linear-based model to an RNN-based model, resulting in similar or improved performance with less calibration time.
