# Supplementary material for: Long-term independent use of an intracortical brain–computer interface for speech and cursor control
Source: Nat Med. 2026 Jun 15;32(7):2504–10. doi: 10.1038/s41591-026-04414-6 (PMC13375540; doi:10.1038/s41591-026-04414-6)
Supplement: Supplementary file 3 — Peer Review File [file 41591_2026_4414_MOESM3_ESM.pdf]

nature portfolio

Peer Review File

Long-term independent use of an intracortical brain-computer interface for speech and cursor control

Corresponding Author: Dr Nicholas Card

This file contains all reviewer reports in order by version, followed by all author rebuttals in order by version.

Version 0:

Reviewer comments:

Reviewer #1

(Remarks to the Author)
This study presents an impressive demonstration of one human subject using a multi-modal (intracortial/gaze-based) BCI system for communication and control in the long-term independent use setting. The BCI system is based on decoding speech-related unit activity from four 64-channel Utah arrays placed in speech motor cortex. The BCI system utilizes a recurrent neural network (RNN)-based decoder with an extensive vocabulary of 125,000 words. The decoder first translates neural features into phoneme probabilities and then uses language modelling to form the most likely word sequence, which is then synthesized into the subject's own voice. To optimize the signal-to-noise of the produced neural features, the study evaluated different "speaking" strategies, which demonstrated a marked increase in the communication performance when using silent speech over vocalized speech. The study subject was a patient with ALS suffering from severe paralysis and dysarthria. After the first 9 months, the subject transitioned from controlled use of the device during 2-4 weekly sessions to independent use, which required only minimal daily calibration. During this period, performance remained stable, and use of the system markedly increased. BCI communication performance reached an average of 56 words per minute, with 92% of decoded sentences labeled by the subject as mostly correct. The BCI system enabled the subject to activate privacy mode (via auxiliary control) to mark data that would remain protected from analysis, ensuring the subject's full control over the use of their data. Auxiliary control functions necessary for independent use and to classify the correctness of the decoded sentences are based on monitoring the subject's gaze. As gaze control may fade in the progression of ALS, the system also provided BCI-based cursor control functionality to the subject. Although Utah array placement was not optimized for motor kinematic decoding, the cursor control was sufficient for 2-dimensional control of a cursor and selecting auxiliary control functions. Overall, this is a well-conceived study, that was executed with high scientific rigor, summarized in a well-written and well-illustrated manuscript that presents an impressive and highly successful body of work. In addition to the impressive technical contributions, the manuscript also presents significant new neuroscientific insights gained from the long-term use of a multi-modal intracortical BCI. For example, insights into alertness (as measured by gaze in this study) will be invaluable in further optimizing BCIs for communication and control. The impressively successful study, along with the unique scientific insight gained from the thoughtful analysis, will be of great interest to the readers of this journal. Overall, there are only a few minor issues and clarifications that I would like the authors to address before the manuscript can be considered ready for publication.

Reviewer #2

(Remarks to the Author)
Summary of major contributions:

This paper addresses two key challenges for deploying intracortical BCIs in the community. The first challenge is providing access to BCI usage without direct or continuous supervision from expert technicians/researchers. The second is maintaining high performance decoding without frequent recalibration. The authors demonstrated both capabilities in a single participant with late-stage ALS.

Detailed Comments:

- As I was reading through the results sections I was wondering how for the 'corrected by user' condition, on average, how

many words in a sentence are corrected? (Or what percentage of words, or how many words per sentence length)

- It would be helpful to understand the importance of eye-gaze control in this setup. How many times per session and what fraction of time per session did the user require gaze control?

- What were the criteria for labeling a word as incorrect? If it is plural instead of singular, is it wrong? Or does the whole word have to be incorrect?

- Compared to the other figures in this paper, Figure 3 is a bit sloppy. The font sizes vary widely and the y-axes are unlabeled in panels B-E.

- In the limitations, I think it should be more clear that this requires a large four computer rack mounted system that is not portable. The network configuration is described clearly in the Methods section, but the bulkiness of the system should be mentioned as a limitation. Another limitation is the reliance on eye-gaze control, which often fails in people with “late-stage ALS”.

- It would be helpful to understand more about the user and caregiver perspectives, particularly with regards to ease-of-use, training time, and impact on daily life. Clearly, from the amount and duration of use, the participant is deriving significant value from the system.

- I personally like Figure S4 and would recommend it be included in the main text

- Figure S5 - add units to spike band power in x-axis labels (or a.u. if arbitrary). Also, panels A and B titles both say "sentences". Does this mean that sentences were produced by the decoder while the participant was asleep? Please clarify

- How was asleep versus tired determined? Was there an objective measure of ‘tired’ or sleepiness?

- How accurate is the eye tracking data? Was it/would it be sustainably impacted by external factors (such as lighting conditions)?

Reviewer #3

(Remarks to the Author)
This manuscript describes long-term (19 months) performance of combined speech and cursor decoding from a BrainGate2 clinical trial participant. The system demonstrated here maintained robust performance over more than two years, albeit with updates to the underlying decoding models. Crucially, the authors demonstrated that the clinical-trial participant could use the system with no assistance from researchers other than periodic updates to the decoders and software, requiring only assistance from a caregiver to connect the BCI and turn on the hardware, which usually required about 20 minutes. The participant used the system as his primary mode of communication and control of his computer to accomplish both professional and personal tasks. Cursor control required a total of only 10 minutes of calibration throughout the day. Interestingly, both speech and cursor/click control were decoded from the same neural signals recorded in speech motor cortex. Moreover, the authors present compelling data on the stability of the neural signals over the course of the study. This report should be of great interest to a broad scientific audience. Although it does not present a major new scientific advance, it does make a substantial contribution to the burgeoning field of intracortical BCI’s by demonstrating the feasibility, stability, and utility of long-term high performance multimodal intracortical BCI usage. This represents an important translational advance that is anticipated to have a significant impact on the field.
Major comments:
1. The manuscript could better distinguish the results of this work and those of the results from the authors’ prior published works. This is not to diminish the novelty of this paper, which is certainly strong, but more an encouragement to clearly define what aspects of research have already been reported and have not, especially in the Introduction section. For speech decoding, if the authors reported the results from the same model and same system as reported in ref. [5], then the little-to-no daily calibration and high decoding accuracy aspect have already been published. The authors should consider clarifying that the novelty comes from long-term usage and results, as well as multi-modal cursor and speech decoding.
2. Was the shift from the original RNN-based model to transformer on Day 600 a sudden switch or was the participant given an opportunity to practice and adapt to the new model? It may be surprising that the shift to a completely new model did not cause any temporary drop in performance. If this is true, can the authors offer any explanation, however, hypothetical, as to why?
3. The calibration time described in the cursor decoding session for both the old and new model seem to be substantial enough for the participant to have noticed. Is the continued calibration throughout the day initiated by the user or the decoding pipeline? sd
4. Figure 4C. Day 25 modulation map for 55b array looks very different from the ones later on. Can the authors explain why? Also, how did the authors choose the four dates? They are not evenly spaced.
5. I think it would be beneficial to also include impedance measurements in the neural signal stability section.
6. For the click decoder, since the participant could adjust the sensitivity, what is the average T throughout the reporting period and its distribution?
7. Given the fact that the decoding accuracy evaluation was based on self-reports, did the authors attempt to measure accuracy from self-ratings?
Minor comments:

8. At least one other study (Vansteensel et al. 2016) has reported independent home use of a BCI by a participant with ALS. I believe the same authors also reported their signal stability over multiple years.
9. Figure 1B. What is the gesture being used in the click decoder?
10. Figure 1E. What is the Other category?
11. Some of the imprecise terms could be replaced or followed by concrete numbers if the information is not otherwise provided in the manuscript. Examples include:
'…used the system in his home [nearly every day] for 19 months…' What is the exact frequency?
'requiring [little-to-no] daily calibration' What does 'little' mean here?
12. Figure 2 B and C: It might make sense to use Day 600 as another cutoff point since a new model was used. It could be beneficial to add two more arrows to Figure 2A indicating Day 412 and Day 600.
13. I think some context is missing in the neural signal stability section. It would be nice if the authors could provide this context and explain (or theorize) how this study was able to overcome presumed instability.
14. It does seem like the overall operation of the system still requires a small dependency on eye tracking. If this is indeed the case, the authors should make this clearer.

Version 1:

Reviewer comments:

Reviewer #1

(Remarks to the Author)
The reviewer thanks the authors for their thoughtful response to the reviews and for adding the suggested clarifications to the manuscript. The revised manuscript has substantially improved in quality, and there are no remaining issues that would prevent it from publication in its present form.

Reviewer #2

(Remarks to the Author)
Bravo! Thank you for addressing my comments in full. Congratulations on a very exciting and impactful body of work.

Reviewer #3

(Remarks to the Author)
The authors have thoroughly and appropriately addressed all of my comments.

Open Access This Peer Review File is licensed under a Creative Commons Attribution 4.0 International License, which permits use, sharing, adaptation, distribution and reproduction in any medium or format, as long as you give appropriate credit to the original author(s) and the source, provide a link to the Creative Commons license, and indicate if changes were made.

In cases where reviewers are anonymous, credit should be given to 'Anonymous Referee' and the source.
The images or other third party material in this Peer Review File are included in the article's Creative Commons license, unless indicated otherwise in a credit line to the material. If material is not included in the article's Creative Commons license and your intended use is not permitted by statutory regulation or exceeds the permitted use, you will need to obtain permission directly from the copyright holder.
To view a copy of this license, visit https://creativecommons.org/licenses/by/4.0/

Response to reviewers, “Long-term independent use of an intracortical brain-computer interface for speech and cursor control” (NMED-A143760)

Editor remarks

Dear Dr. Card,

Your Article, "Long-term independent use of an intracortical brain-computer interface for speech and cursor control" has now been seen by 3 referees. You will see from their comments below that while they find your work of interest, some important points are raised. We are very interested in the possibility of publishing your study in Nature Medicine but would like to consider your response to these concerns in the form of a revised manuscript before we make a final decision on publication.

We therefore invite you to revise your manuscript taking into account all reviewer and editor comments. Please highlight all changes in the manuscript text file.

We sincerely thank the editor and reviewers for their time and effort providing feedback on this manuscript. We are heartened that the reviewers had such positive comments about our work:

> *“Overall, this is a well-conceived study, that was executed with high scientific rigor, summarized in a well-written and well-illustrated manuscript that presents an impressive and highly successful body of work.”* - Reviewer 1
>
> *“This paper addresses two key challenges for deploying intracortical BCIs in the community.”* - Reviewer 2
>
> *“This represents an important translational advance that is anticipated to have a significant impact on the field.”* - Reviewer 3

We have gone through the feedback from each reviewer and responded point-by-point with clarifications, additional analyses/figures, and changes to the text, where appropriate. We have also included several new videos that show the participant using various features of the BCI system.

In this Response to Reviewers document, the original reviewer comments appear in black text. Our responses to the reviewers appear in green text. When we quote updated passages of text from the revised manuscript, they appear in

> indented black text in which the relevant changed portions of text appear in red. If the quoted passage includes changes made in response to different reviewer concerns or are minor unrelated changes for clarity, these changes appear in dark red to more clearly maintain emphasis on those changes that address this particular reviewer concern.

Reviewer #1 (Remarks to the Author):

This study presents an impressive demonstration of one human subject using a multi-modal (intracortial/gaze-based) BCI system for communication and control in the long-term independent use setting. The BCI system is based on decoding speech-related unit activity from four 64-channel Utah arrays placed in speech motor cortex. The BCI system utilizes a recurrent neural network (RNN)-based decoder with an extensive vocabulary of 125,000 words. The decoder first translates neural features into phoneme probabilities and then uses language modelling to form the most likely word sequence, which is then synthesized into the subject's own voice. To optimize the signal-to-noise of the produced neural features, the study evaluated different "speaking" strategies, which demonstrated a marked increase in the communication performance when using silent speech over vocalized speech. The study subject was a patient with ALS suffering from severe paralysis and dysarthria. After the first 9 months, the subject transitioned from controlled use of the device during 2-4 weekly sessions to independent use, which required only minimal daily calibration. During this period, performance remained stable, and use of the system markedly increased. BCI communication performance reached an average of 56 words per minute, with 92% of decoded sentences labeled by the subject as mostly correct. The BCI system enabled the subject to activate privacy mode (via auxiliary control) to mark data that would remain protected from analysis, ensuring the subject's full control over the use of their data. Auxiliary control functions necessary for independent use and to classify the correctness of the decoded sentences are based on monitoring the subject's gaze. As gaze control may fade in the progression of ALS, the system also provided BCI-based cursor control functionality to the subject. Although Utah array placement was not optimized for motor kinematic decoding, the cursor control was sufficient for 2-dimensional control of a cursor and selecting auxiliary control functions. Overall, this is a well-conceived study, that was executed with high scientific rigor, summarized in a well-written and well-illustrated manuscript that presents an impressive and highly successful body of work. In addition to the impressive technical contributions, the manuscript also presents significant new neuroscientific insights gained from the long-term use of a multi-modal intracortical BCI. For example, insights into alertness (as measured by gaze in this study) will be invaluable in further optimizing BCIs for communication and control. The impressively successful study, along with the unique scientific insight gained from the thoughtful analysis, will be of great interest to the readers of this journal. Overall, there are only a few minor issues and clarifications that I would like the authors to address before the manuscript can be considered ready for publication.

Minor Issues:

1. Why is silent speech more effective than overt speech?

   To clarify, we observed a tradeoff of communication speed and decoding accuracy between the attempted silent and attempted vocalized speaking strategies. Participant T15 can physically attempt to speak at a faster rate using the silent speaking strategy than he can when using the vocalized speaking strategy (Figure 2E, bottom), but the decoding accuracy during attempted silent speech is slightly worse than it is during attempted vocalized speech (Figure 2E, top). T15 has told us that attempted vocalization

is more effortful and energy-draining for him than attempted silent speech due to his ALS. We speculate that during the more effortful vocalization, he attempts to move his articulators (and vocalizes) more strongly and more precisely, which we would predict might be accompanied by higher signal-to-noise ratio neural modulation. Based on our approach to decoding, this would lead to slightly higher decoding accuracy. T15 himself chooses to speak silently, since it's less effortful and our custom GUI enables him to correct decoding errors during personal use.

We have now clarified in the Results section that the silent speaking strategy was less fatiguing for the participant:

> We did not instruct T15 to use a particular speaking strategy during personal use of the system; instead, he was encouraged to use whichever approach felt most sustainable, natural, and effective. The opportunity to develop a strategy that minimizes user fatigue was especially important, given the known challenges of fatigue in patients with late-stage ALS[23]. Initially, he employed attempted "vocalized speech", producing sound while speaking. Over time, he transitioned to a "silent speech" strategy – making facial muscle movements without phonating – which he noted was less effortful for him. This strategy change was associated with an increase in speaking speed, from ~30 to over 50 words per minute (WPM; Fig. 2A, subplot 2). To benchmark longitudinal performance of the system, and to quantify the effect of silent vs. overt speaking strategies, we periodically asked him to repeat sentences presented on a screen in a Copy Task (Fig. 2E). During vocalized speech, accuracy exceeded 99% at 30.6 WPM average; with silent speech, accuracy reached 96.5% at 49.7 WPM average. This difference in decoding accuracy may be attributable to differences in neural modulation between the attempted vocalized and silent speaking strategies (Fig. S7). Note that personal use and benchmark sessions after post-implant day 600 utilized a more accurate transformer-based phoneme decoding architecture, while prior sessions used a recurrent neural network-based model (Fig. S2).

We have also added an additional supplemental figure Fig. S7 (referenced in the updated text, above) that compares neural modulation depth between attempted vocalized and silent speech. We analyzed data from 8 research sessions that included data samples of *both* attempted vocalized and attempted speech (n=1857 attempted vocalized trials, n=1008 attempted silent trials). We found that 55b was less modulated during vocalized trials than silent trials, and d6v displayed the opposite trend. The M1 and v6v arrays had mixed trends, although there does appear to be some spatial organization to this mixture.

Future research projects and analyses may seek to further examine differences in neural modulation between speaking strategies, which may necessitate recording additional controlled datasets wherein the participant repeats prompted words or phrases with a range of speaking strategies on the same day(s).

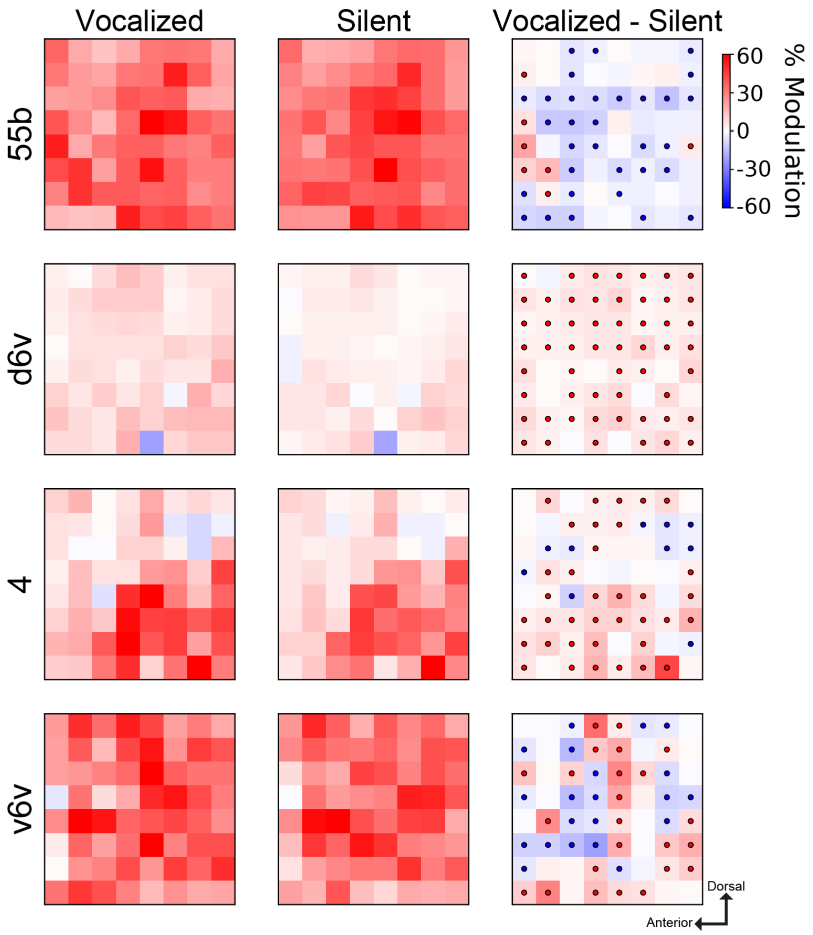

Figure S7. Neural modulation during attempted vocalized or silent speech. Spike band power modulation for each microelectrode on each array during attempted vocalized or silent speech. Data is sourced from 8 research sessions containing both speaking strategies and includes n=1857 trials of attempted vocalized speaking and n=1008 trials of attempted silent speaking. Consistent with Fig. 4C, modulation depth for each electrode is calculated as the average change in spike band power between speaking epochs and non-speaking epochs for each trial. In the third column, a 2-sample t-test (alpha = 0.05 / 256) is used to compare the distributions of modulation depth for attempted vocalized and attempted silent speaking trials. Red circles indicate that attempted vocalized speaking had significantly more neural modulation than attempted silent speaking. Blue circles indicate that attempted silent speaking had significantly more modulation than attempted vocalized speaking.

2. What happened during the days when the patient was not using the BCI?

   Prior to post-implant day 281, before independent use of the BCI was enabled, our IDE restricted T15 to use the BCI only with a member of our research team present, which

for practical reasons was limited to 2-4 days per week. Starting at post-implant day 281, when independent use of the BCI was enabled and our IDE was granted a Humanitarian Exemption, T15 and his care partners could use the BCI whenever they wanted, and we left it up to them to decide when to use it. From then on, days where the BCI was *not* used might include days where the participant was not at home (e.g., traveling) or just chose not to use the system for some reason (which we treated as his personal information and hence did not document). We have added a note to the Results to explain this:

> We previously quantified T15's natural speech and effective communication rates with his preferred AAC device[5]. Rather than relying on his existing communication strategies, T15 chose the BCI as his primary mode of communication and computer interface. He used it to converse with family, friends, colleagues, and clinicians – in person, over video calls, and through digital applications such as email and text messaging. He also used it for both professional and recreational computer access. Before post-implant day 281, usage was limited to 2-4 weekly sessions requiring a research assistant (author C.I.) to oversee setup and use (3.7 hours/day average). After the investigational device exemption (IDE) was modified allowing his care partners to don and doff the system without the scientific team being present on day 281, T15 and his care partners could initiate system use independently at their home whenever they wanted, resulting in a marked increase in usage to 9.5 hours/day average (Fig. 1D). As of 678 days after the implant surgery, he had accumulated over 3,800 hours of BCI use in 444 days (Fig. 1E). The participant used the BCI at his own discretion. Days where the BCI was *not* used may have happened for a variety of private reasons (e.g., the participant was traveling or simply chose not to use the system).

3. What is the decoding latency? How well would this be suitable for real-time conversations?

   We have added details about system latency during speaking epochs and sentence finalization to the Methods section of the manuscript:

> <u>Speech decoding latency</u>
> Decoding latency for the speech decoder can be separated into two categories: (1) real-time feedback during attempted speech and (2) sentence finalization.
>
> During attempted speech, there were two primary sources of decoding latency. The first is the use of an acausal Gaussian smoothing strategy, which introduced 160 ms of latency. The second was the patching strategy for inputting neural features into the decoder itself. Neural features were grouped into optionally-overlapping chunks and fed into the speech decoder every 80 ms.

These two sources combined accounted for the majority of the system latency during real-time inference, estimated to be 160-240 ms. Phoneme decoder inference and language model inference typically took less than 1 ms each.

At the end of a sentence, there was additional latency introduced from sentence finalization, which included (1) re-normalizing and re-smoothing the neural features from the entire speaking epoch with updated normalization statistics, (2) putting the reprocessed neural features through the phoneme encoder to get updated phoneme logits, (3) sending the updated phoneme logits through the language model, (4) finalizing the language model with a larger and more accurate unpruned ngram model to obtain the top 100 most likely word sequences, and (5) rescoring these candidate word sequences with a large language model. Between each of these steps, there may potentially be some additional latency introduced by local network communication between individual nodes via the redis server.

We performed a comprehensive analysis of sentence finalization latency throughout our study (Fig. S9). We grouped the latency into two stages: phoneme encoder latency, which includes steps 1-2 above, and language model latency, which includes steps 3-5 above. We found that the RNN-based phoneme encoder took a median of 772 ms to finalize, and these RNN-predicted phoneme logits took a median of 1921 ms to finalize into the most likely word sequence, for a total median finalization latency of 2657 ms (Fig. S9A). The transformer-based phoneme encoder took a median of 85 ms to finalize, and these transformer-predicted phoneme logits took a median of 1623 ms to finalize into the most likely word sequence, for a total median finalization latency of 1791 ms (Fig. S9B). We note that although the language model pipeline was the same irrespective of the phoneme encoder model type, it likely had lower latency when paired with the higher-accuracy transformer phoneme encoder because more-accurately-predicted phoneme logits necessitated a less-exhaustive beam search at the language model stage. Finally, we note that these latencies scaled with the number of words in the final decoded sentence (Fig. S9C). Shorter sentences took less time to finalize, while longer sentences took more time. Sentences under 20 words long took 2072 or 1171 median total ms (RNN and transformer, respectively) to finalize, whereas sentences 20 words or longer took 6258 or 5181 median total ms.

And we have added a new supplemental figure detailing the sentence finalization latency:

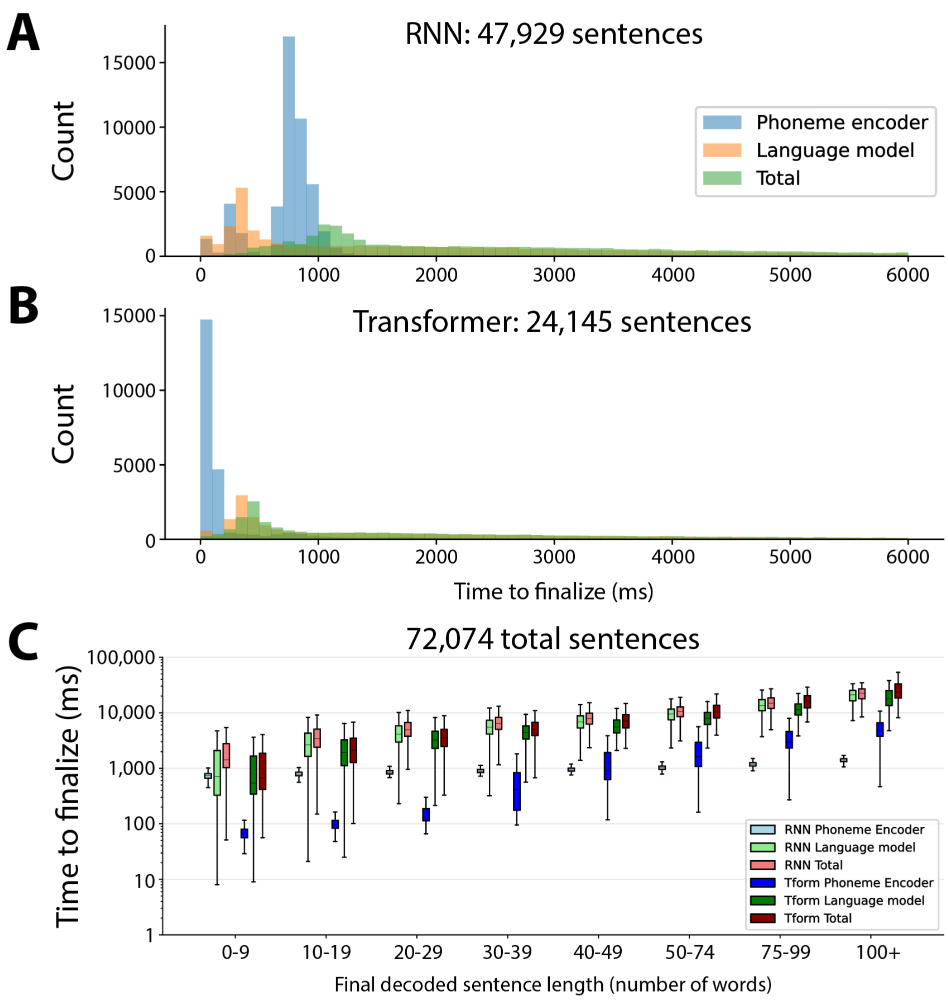

Figure S9. Sentence finalization latency. (A) Distribution of sentence finalization latencies across 47,929 personal use sentences using the RNN-based phoneme encoder. **(B)** Distribution of sentence finalization latencies across 24,145 personal use sentences using the transformer-based phoneme encoder. **(C)** Sentence finalization latencies presented as a function of the number of words in the final decoded sentence.

4. The performance reporting for mostly correct sentences, especially considering the self-reporting of the correctness of the decoded sentences, deserves a more detailed presentation. A few examples of such sentences (correct vs. mostly-correct) would be helpful to understand the intelligibility of the produced sentences.

   Thank you, and we agree. We have added examples of personal use decoded sentences that T15 rated as “correct”, “one word wrong”, “mostly correct”, or “incorrect” to supplemental table S3, which we share with his permission:

   Table S3. Example decoded sentences for each accuracy rating.*

**Rating**	**Decoded sentence**
Correct	he wants a work plan before he leaves to go back to australia
Correct	step number six is the mastery of the skill and the ability to navigate emergency situations
Correct	can you turn on the air conditioning i am getting hot
One word wrong	thank you and i will have this later
One word wrong	and the computer is taking that attention and turning it into words on the screen in front of me
One word wrong	if not no worries but i will need to know today because it will require us to modify our classes to be ready in time
Mostly correct	thank you i am expecting other news i am simply assessing my other needs
Mostly correct	very decision because you will see so many jokes
Mostly correct	he was also creating a video game for killer whales
Incorrect	we were to understand or figure out in that moment
Incorrect	that is not what i am talking about i want to get access to the premium waters for the anna doll
Incorrect	it is more of a concern for men in here i was just looking for a general category that a button or a light one or i am a big deal

* selected personal use transcripts shared with participant's permission

5. Reporting days and months of use interchangeably is confusing for the reader, especially in Figure 4. I suggest reporting use in months consistently.

Thank you for this feedback. When referring to data spanning large time periods, we use the "months" term. However, when referring to individual days of data, we think it is more clear to use the specific post-implant day number(s) (e.g., post-implant day 25). We have now edited the text throughout the document to stick to this convention consistently. Below are two examples of this type of edit:

In the Introduction:

> We previously quantified T15's natural speech and effective communication rates with his preferred AAC device[5]. Rather than relying on his existing communication strategies, T15 chose the BCI as his primary mode of communication and computer interface. He used it to converse with family,

friends, colleagues, and clinicians – in person, over video calls, and through digital applications such as email and text messaging. He also used it for both professional and recreational computer access. Before post-implant day 281, usage was limited to 2-4 weekly sessions requiring a research assistant (author C.I.) to oversee setup and use (3.7 hours/day average). After the investigational device exemption (IDE) was modified allowing his care partners to don and doff the system without the scientific team being present on day 281, T15 and his care partners could initiate system use independently at their home whenever they wanted, resulting in a marked increase in usage to 9.5 hours/day average (Fig. 1D). As of ~~678 days~~ 22.6 months after the implant surgery, he had accumulated over 3,800 hours of BCI use, and he used the BCI on 444/653 days (Fig. 1E). The participant used the BCI at his own discretion. Days where the BCI was not used may have happened for a variety of private reasons (e.g., the participant was traveling or simply chose not to use the system).

And in the Results:

To assess the stability of task-relevant neural modulation, we measured the change in spike band power between rest and speech epochs for each electrode on each day. These speech modulation maps appeared qualitatively stable across ~~hundreds of days~~ months (Fig. 4C). We quantified this by computing the cosine similarity between daily neural modulation vectors for each array as a function of time separation (Fig. 4D). Cosine similarities for all arrays – except the d6v array, which did not exhibit much speech-related modulation – remained above 0.6 even for comparisons separated by more than 18 months, demonstrating that task-relevant neural representations remained quite stable for at least 19 months.

6. When the time of separation is picked in Figure 4 remains unclear.

Thanks for the feedback. To clarify, panels 4A, 4B, and 4D include data from all days of BCI use. The specific days chosen for Figure 4C were arbitrarily chosen to roughly uniformly span the post-implantation period (i.e., post-implant day 25 to post-implant day 678). In response to this comment, we have now adjusted panel 4C to more precisely uniformly span this period. We now show neural modulation on days 25, 242, 460, and 678 – 217-218 days between each timepoint. We have also amended the figure caption to explain this rationale:

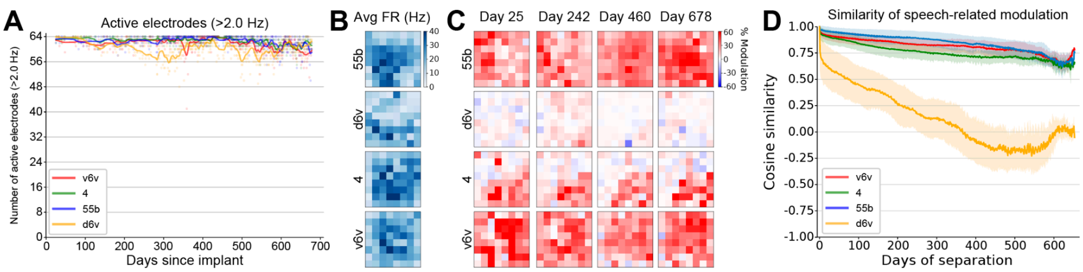

Figure 4. Neural stability. (A) Number of electrodes per array with firing rates of 2 Hz or greater during speaking epochs, plotted by post-implant day. Dots are individual data points and lines are Gaussian-smoothed approximations. Colors correspond to the four arrays. **(B)** Average firing rate for each electrode during speaking epochs from the entire 444-session dataset. Electrode locations are drawn according to their physical location on each of the 4 arrays, where up corresponds to dorsal and left corresponds to rostral. **(C)** Speech-related neural modulation for each electrode on a given example day, expressed as the percentage change in spike band power between rest epochs and speaking epochs. Each column corresponds to the post-implant day indicated by the column title. Days were chosen to uniformly span the post-implant period (25-678 days post-implant). **(D)** Cosine similarity between daily speech-related neural modulation vectors, as a function of the number of days between each pair. Colors correspond to the four arrays. Solid lines are means and shaded regions represent one standard deviation around the mean.

7. A more direct comparison of the stability and performance between gaze-based and BCI-based cursor control would be helpful.

T15 was given the option to switch at will between gaze-based and BCI-based cursor control for navigation of the BCI user interface. BCI-based cursor control could also be used to control his own personal computer. During nearly all independent use, he chose to use gaze-based control for the BCI user interface while reserving BCI-based cursor control for use with his own personal computer.

We have edited the Figure 1 caption to emphasize this point:

Figure 1. Independent use of the multimodal intracortical BCI. (A) Schematic of the participant using the multimodal intracortical BCI to control their personal computer. As the participant tried to speak, his neural activity was decoded into words on a screen. He could also control his computer by attempting to move or squeeze his hand. The system also integrated eye tracking to enable the participant to select on-screen buttons by looking at them for a short time. The participant could switch between gaze-based and neural cursor-based control of the user interface by selecting his preference through the custom-made GUI. (B) Locations of four 64-microelectrode arrays in the participant's dominant ventral

precentral gyrus (left). The dotted line is the dominant central sulcus. Each electrode records activity from individual neurons and local field potentials. Recorded neural signals were processed into binned sequences of neural features (middle) and inputted to speech, cursor, or gesture decoders to decode the user's intended words, cursor movements, or clicks (right). (C) User interface examples during speech decoding (left), sentence rating (middle), and sentence correction (right). (D) Cumulative hours of system usage over time. Independent use began at post-implant day 281. (E) Time distribution of how the system was used across 3,801 hours sourced from all personal use sessions. "Other" refers to idle time where the BCI system was active but the participant was not using it.

We also edited the the *Cursor decoding* subsection of the Results:

T15 configured and toggled cursor control through the BCI's gaze-based interface, which allowed him to select on-screen buttons to start or stop control, paste recent decoded sentences, and issue function key presses (e.g., escape key; Fig. S6). He could also use the neural cursor to navigate the BCI user interface (Video 11), though he typically stuck to gaze-based control there while reserving the neural cursor for his personal computer. Each day, he calibrated the cursor and click decoders via a short calibration routine[17,18]. For calibration we used a center-out-and-back target acquisition task with fixed target distance and size until day 621, after which we used a more difficult task with varied target positions and smaller target sizes (see Methods).

To directly compare the performance of neural cursor control and gaze-based cursor control, we collected a new dataset where T15 was asked to perform a grid task using gaze-based cursor control for direct comparisons with previously-collected neural cursor grid task data. With a 14x14 grid (each square measures 0.77 x 0.77 inches on screen), T15 achieved 0.80 ± 0.33 (mean ± standard deviation) bits per second (BPS) with eye gaze control, and 2.90 ± 0.16 BPS with neural cursor control. On a 6x6 grid with larger grid tiles (1.79 x 1.79 inch squares), T15 made selections at 1.67 ± 0.21 BPS with neural cursor and 2.59 ± 0.26 BPS with gaze control. We have added these numbers to the Methods section:

Eye tracking

T15's eye gaze was tracked using a Tobii Pro Spark eye tracker (Tobii AB, Stockholm, Sweden) that was mounted at the bottom of the BCI system participant monitor. Calibration was performed at the start of each session (for both research sessions and independent sessions) and repeated as needed via an option available in the BCI system menu (Video 13). Gaze control typically required one calibration routine at the beginning of each day, plus potential additional recalibrations if T15 moved, if the monitor was moved, or if lighting conditions changed. The gaze calibration routine took less than 30 seconds and could be accessed at any time through the BCI user interface's menu screen.

Gaze location was sampled at 60 Hz, averaged across both eyes, smoothed over time, and used to enable gaze-based selection of on-screen buttons. Button activation was triggered by maintaining gaze on an on-screen target for 0.5-1.0 seconds.

To compare T15's neural cursor control with gaze-based control, we asked him to perform the same grid task (14 x 14 grid where each tile measures 0.77 x 0.77 inches on screen) using gaze-based control. His bit rate was 0.80 ± 0.33 bits per second. With larger grid tiles (6 x 6 grid, 1.79 x 1.79 inches per tile), T15 made selections at 1.67 ± 0.21 bits per second with neural cursor and 2.59 ± 0.26 bits per second with gaze control.

To improve usability and reduce selection errors, we implemented a custom "magnetization" feature that subtly attracted the gaze cursor to the center of nearby on-screen buttons, making them easier to select. Eye tracker calibration, gaze data acquisition, and selection logic were implemented in custom Python software integrated into the BRAND-based data collection platform.

8. The supplementary videos should be clearly annotated to indicate when gaze and when BCI-based cursor control was used.

Thank you for this helpful suggestion. We have updated all of the videos included with this manuscript, and now clearly describe in each video caption when T15 is using gaze-based or BCI-based cursor control.

For example:

Video 1. T15 describes the impact of the BCI on his life. T15 uses the speech BCI to describe how the system impacts his everyday life. He is navigating the user interface with eye gaze, which is rendered on-screen as a semi-transparent white circle. His attempted speech is automatically detected by the speech decoder, and the decoded words are displayed on-screen in real time. The BCI system monitor is on an articulating monitor arm, which is mounted on his desk and positioned above his personal computer screens. This video was recorded on post-implant day 678.

Video 11. Navigating the BCI user interface with neural cursor control. T15 first uses eye gaze to enable neural cursor control. When neural cursor control is enabled, a mouse cursor is rendered on the screen, and when T15 attempts to click, a growing blue circle appears at the tip of the cursor. Note that, even when neural cursor control is enabled, T15's eye gaze is still rendered on the screen as a semi-transparent white circle, although it is not used for actually navigating the user interface. This video was recorded on post-implant day 874.

<u>Reviewer #2 (Remarks to the Author):</u>

Summary of major contributions:

This paper addresses two key challenges for deploying intracortical BCIs in the community. The first challenge is providing access to BCI usage without direct or continuous supervision from expert technicians/researchers. The second is maintaining high performance decoding without frequent recalibration. The authors demonstrated both capabilities in a single participant with late-stage ALS.

Detailed Comments:

1. As I was reading through the results sections I was wondering how for the 'corrected by user' condition, on average, how many words in a sentence are corrected? (Or what percentage of words, or how many words per sentence length)

Thank you for noting this opportunity to provide additional useful details about the real-world usage of the system. Through post-implant day 678, T15 made corrections to 23,566 total sentences before rating them as "100% correct". While making corrections, T15 changed (inserted, deleted, or substituted) 31,557 out of 248,586 total words (12.7%). The corrected sentences were 10.50 ± 9.19 (mean ± standard deviation) words long and had 1.33 ± 0.65 corrected words per sentence. We have added a subset of these details to the Results text:

> Decoding accuracy was self-rated by T15 after each sentence and remained relatively stable over months (Fig. 2A, top). The ability to make corrections using the custom-built graphical user interface, controlled via gaze or cursor, became available at post-implant day 227. We observed that sentence accuracy was influenced by multiple factors, including fatigue (Fig. S5), attempted speaking rate (Fig. 2A, subplot 2), sentence length (Fig. 2A, subplot 3), and topic. System updates – including new feature implementations, bug fixes, and decoder upgrades – also contributed to performance variability (Fig. S2). Across 183,060 sentences, 53.3% were decoded completely correctly, 12.9% were corrected by the participant, and 26.1% were mostly correct (Fig. 2B). Of the 23,566 sentences that T15 corrected via the user interface, 12.7% of words (31,557 / 248,586) were changed during the correction process. The overall trend of decoding performance improved over time, with later months showing a higher proportion of correct sentences (rank sum test; p<0.001; Fig. 2C) and increased sentence length (p<0.001; Fig. 2A, subplot 3). We note that T15 often strung multiple sentences together at a time into a single utterance – the longest correctly decoded utterance was 215 words (Fig. S4) – which had the effect of lowering the self-rated accuracies (as an utterance gets longer, the probability of it being decoded 100% correct decreases).

2. It would be helpful to understand the importance of eye-gaze control in this setup. How many times per session and what fraction of time per session did the user require gaze control?

T15 was given the option to switch at will between gaze-based and BCI-based cursor control for navigation of the BCI user interface. He almost always chose to use gaze-based control for the BCI user interface while reserving neural cursor control for his personal computer. Gaze control typically required one calibration routine at the beginning of each day, plus potential additional recalibrations if T15 moved, if the monitor was moved, or if lighting conditions changed. The gaze calibration routine took less than 30 seconds and could be accessed at any time through the BCI user interface's menu screen.

We have updated the Figure 1 caption text to explain this:

> **Figure 1. Independent use of the multimodal intracortical BCI.** (A) Schematic of the participant using the multimodal intracortical BCI to control their personal computer. As the participant tried to speak, his neural activity was decoded into words on a screen. He could also control his computer by attempting to move or squeeze his hand. The system also integrated eye tracking to enable the participant to select on-screen buttons by looking at them for a short time. The participant could switch between gaze-based and neural cursor-based control of the user interface by selecting his preference through the custom-made GUI. (B) Locations of four 64-microelectrode arrays in the participant's dominant ventral precentral gyrus (left). The dotted line is the dominant central sulcus. Each electrode records activity from individual neurons and local field potentials. Recorded neural signals were processed into binned sequences of neural features (middle) and inputted to speech, cursor, or gesture decoders to decode the user's intended words, cursor movements, or clicks (right). (C) User interface examples during speech decoding (left), sentence rating (middle), and sentence correction (right). (D) Cumulative hours of system usage over time. Independent use began at post-implant day 281. (E) Time distribution of how the system was used across 3,801 hours sourced from all personal use sessions. "Other" refers to idle time where the BCI system was active but the participant was not using it.

And in the *Cursor decoding* subsection of the Results:

> T15 configured and toggled cursor control through the BCI's gaze-based interface, which allowed him to select on-screen buttons to start or stop control, paste recent decoded sentences, and issue function key presses (e.g., escape key; Fig. S6). He could also use the neural cursor to navigate the BCI user interface (Video 11), though he typically stuck to gaze-based control there while reserving the neural cursor for his personal computer. Each day, he calibrated the cursor and click decoders via a short calibration routine[17,18]. For calibration we used a center-out-and-back target acquisition task with fixed target distance and size until day 621, after which we used a more difficult task with varied target positions and smaller target sizes (see Methods).

And in the *Eye tracking* subsection of the Results:

> T15's eye gaze was tracked using a Tobii Pro Spark eye tracker (Tobii AB, Stockholm, Sweden) that was mounted at the bottom of the BCI system participant monitor. Calibration was performed at the start of each session (for both research sessions and independent sessions) and repeated as needed via an option available in the BCI system menu (Video 13). Gaze control typically required one calibration routine at the beginning of each day, plus potential additional recalibrations if T15 moved, if the monitor was moved, or if lighting conditions changed. The gaze calibration routine took less than 30 seconds and could be accessed at any time through the BCI user interface's menu screen. Gaze location was sampled at 60 Hz, averaged across both eyes, smoothed over time, and used to enable gaze-based selection of on-screen buttons. Button activation was triggered by maintaining gaze on an on-screen target for 0.5-1.0 seconds.

We also note that despite ALS progression, T15 maintained his baseline gaze control throughout this study; thus, since he was already looking at the screen to use the brain-to-text BCI, it was fast and intuitive to use the gaze control. We anticipate that as his ALS progresses his eye motor control may decline, and in that scenario the neural cursor could become more preferred.

3. What were the criteria for labeling a word as incorrect? If it is plural instead of singular, is it wrong? Or does the whole word have to be incorrect?

T15 was instructed to only label words and sentences correct if they exactly matched what he attempted to say. So if he attempted to say "cats" but the decoder predicted "cat", that would be incorrect. We have clarified this point in the Results section:

> The BCI system in T15's home was a set of networked research computers[22] mounted on a mobile cart, located in his bedroom or living room. T15's care partners were trained to safely connect the neural recording equipment and power on the computer system, which was then automatically initialized through custom software. This allowed T15 to operate the BCI without assistance from any member of the research team. T15 and his care partners reported that daily setup took approximately 20 minutes, after which he used the system continuously for up to 19 hours without additional assistance. Once initialized, T15 operated the system independently using neural decoding, supplemented by eye gaze tracking to facilitate user-interface control. The speech decoder software ran continuously in the background and displayed decoded words on-screen in real time (Fig. 1C, left; Video 1). After each utterance, T15 was given the option to make corrections to the decoded text through a custom user interface via gaze or cursor input (Fig. 1C, right). He then rated decoding accuracy with the following options: "correct," "one word wrong," "mostly correct," or "incorrect" (Fig. 1C, middle; Video 2). T15 was

instructed to rate decoded sentence accuracy strictly, such that he would only mark a sentence as “correct” if it was *exactly* what he tried to say, even down to plurality (e.g., “cat” vs “cats”).

4. Compared to the other figures in this paper, Figure 3 is a bit sloppy. The font sizes vary widely and the y-axes are unlabeled in panels B-E.

Thank you for pointing this out. The lack of y-axis labels was deliberate (we felt that the subplot titles succinctly conveyed both the core idea of the analysis and the units) but we recognize that others may not agree with this aesthetic preference and have now changed it to the more common practice of labeling vertical axes. We have also made the font sizes more uniform throughout the figure. Here is the updated figure (caption remains unchanged):

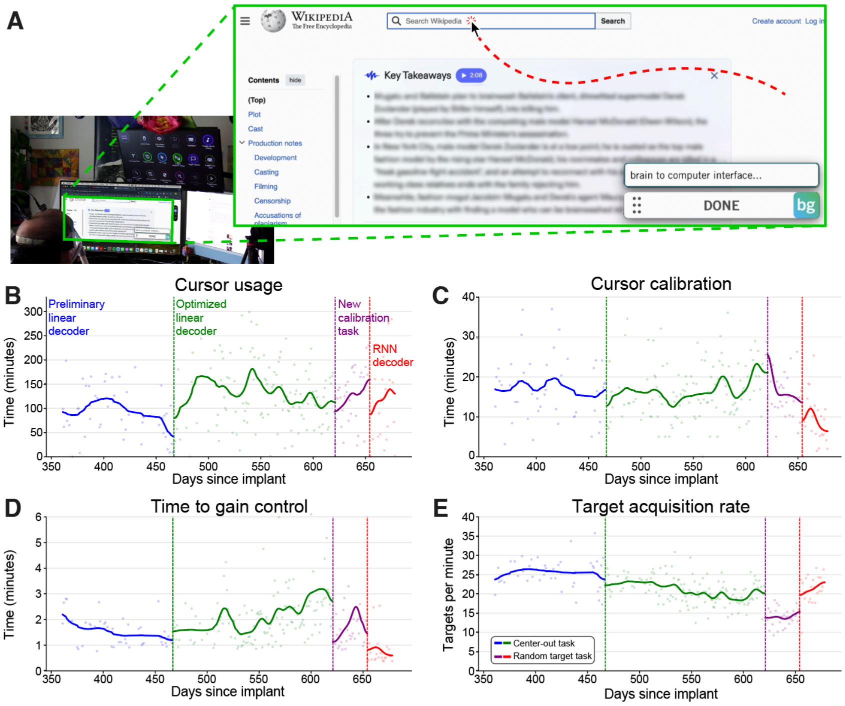

5. In the limitations, I think it should be more clear that this requires a large four computer rack mounted system that is not portable. The network configuration is described clearly in the Methods section, but the bulkiness of the system should be mentioned as a limitation. Another limitation is the reliance on eye-gaze control, which often fails in people with “late-stage ALS”.

The reviewer is correct that this BCI system currently requires a large wheeled computer cart which is not practically portable beyond the confines of the participant’s home. Though on a positive note, the participant has been able to use the system throughout his home and even on his balcony (which we’ve added a mention of since readers might also be interested in this). We also note that the computer system could likely be made substantially more portable, though

that was not the focus of this research and the present multi-computer system facilitates our group's flexible computation needs and need for rapid iteration. We have adjusted the text in the methods section to more clearly address this bulkiness limitation:

> *Data collection rig*
> The BCI system was implemented using a distributed, multi-computer setup designed to support both high-bandwidth neural data collection and low-latency, real-time multimodal neural decoding and user interfaces[22]. While the current configuration includes more computational power and physical footprint than would be needed in a future system suitable for widespread clinical use, it enabled rapid prototyping, flexible experimentation, and continuous decoder optimization. Although this large wheeled computer rack system limits portability, T15 was able to use it throughout his home. In future iterations, the system could be miniaturized into an embedded platform with a portable external interface, similar to prior efforts in reach-and-grasp BCIs using Utah arrays[14].

We have also added a note about portability to the limitations section of the Discussion:

> This study has several limitations. It involved a single participant, and the generalizability of these results to other individuals, electrode implant sites, intracortical electrode types, or neurological conditions is not yet known. The system relied on percutaneous wired connections and required daily setup by trained care partners, which may limit broader adoption. The multi-computer system's bulkiness also limited portability and prevented use outside of the participant's home. We did not systematically assess user fatigue or long-term device wear, although the system's continued high decoding performance suggests durability across the timescales studied. Future work will be needed to evaluate wireless or fully implantable systems, minimize setup time, and expand access to users with different clinical profiles. Finally, speech decoding accuracy achieved during independent conversation was not consistently as high as it was during structured research sessions with prompted sentences. Work remains to deliver a speech neuroprosthesis with the same consistency and accuracy as healthy speech across the full range of what the user is trying to say.

As for gaze control, we note that the system is not currently reliant on gaze control. The entire system can be controlled by BCI-driven cursor control if the participant chooses to do so. But since gaze control works well for him, as described earlier, T15 chooses not to enable this option and sticks with gaze control for navigation of the BCI user interface. If his ALS were to progress to the point where he lost reliable gaze control, we predict that he could still operate the system via BCI-driven cursor control. We have likewise clarified this point in the Methods text, as described in point #2, above.

6. It would be helpful to understand more about the user and caregiver perspectives, particularly with regards to ease-of-use, training time, and impact on daily life. Clearly, from the amount and duration of use, the participant is deriving significant value from the system.

Thank you for this suggestion. We note that a brief description of how the BCI impacts T15's life is available in Video 1. Beyond this, we have gathered feedback from T15 about the usability of the system, which we detail in a separate preprint (Peracha et al., *arXiv* 2026) of a human-computer interface conference proceedings that focuses on the BCI user interface design, the user experience, and the iterative co-design with the user (T15) to customize and improve the user interface to meet his needs. Given that this topic can (and deserves to be) explored a long and in-depth way, but is thematically further removed from the current manuscript's core focus on long-term independent use and decoding accuracy of the BCI, we believe that it is best described in that separate work. Here we have added text to the manuscript to briefly discuss this point and cite that preprint:

> *BCI system software (user interface)*
> We built a custom user interface that supported the daily independent use of the BCI for speech decoding, cursor control, personal computer control, and a variety of related options. The user interface was primarily coded with the Pyglet Python package version 2.0.1237 and consisted of a range of function-specific "pages" that the user could navigate between using gaze or cursor control (Fig. S5). This interface was displayed on a monitor that was mounted on an articulating arm, which was positioned above the participant's personal computer (e.g., see Fig. 3A). The system was periodically updated in response to participant feedback, feature requests, and bug fixes in the underlying custom software stack (Table S2). Additional details about the user interface and T15's feedback on specific design features is available at [41].

We agree with the reviewer that the carepartner's perspective would be a terrific addition to the manuscript. Although our study team has had many informal conversations with T15's care partners, and we can anecdotally say they are very happy with the system, these individuals are not enrolled in the BrainGate2 study and we would not have authorization to publish those conversations even if we had recorded them (which we did not). Given the importance of this question, we hope to update our protocol in the future (or have a separate IRB-approved protocol) to contemporaneously collect this type of care partner feedback in a structured way so that it can be reported in future studies. All of that being said, the current Video 1 does offer a small window into T15's perspective of how the BCI benefits him.

7. I personally like Figure S4 and would recommend it be included in the main text

Thank you for this suggestion. We have now integrated Figure S4 into Figure 2 alongside the other speech decoding results (see below). Alongside this change, we have edited the text accordingly to update figure references. However, we defer to the Editor and are also comfortable reverting and leaving it as a supplementary figure if this makes Figure 2 too

cluttered. We have also made minor additional changes to this figure that were requested by a different reviewer (reviewer 3, minor comment #5).

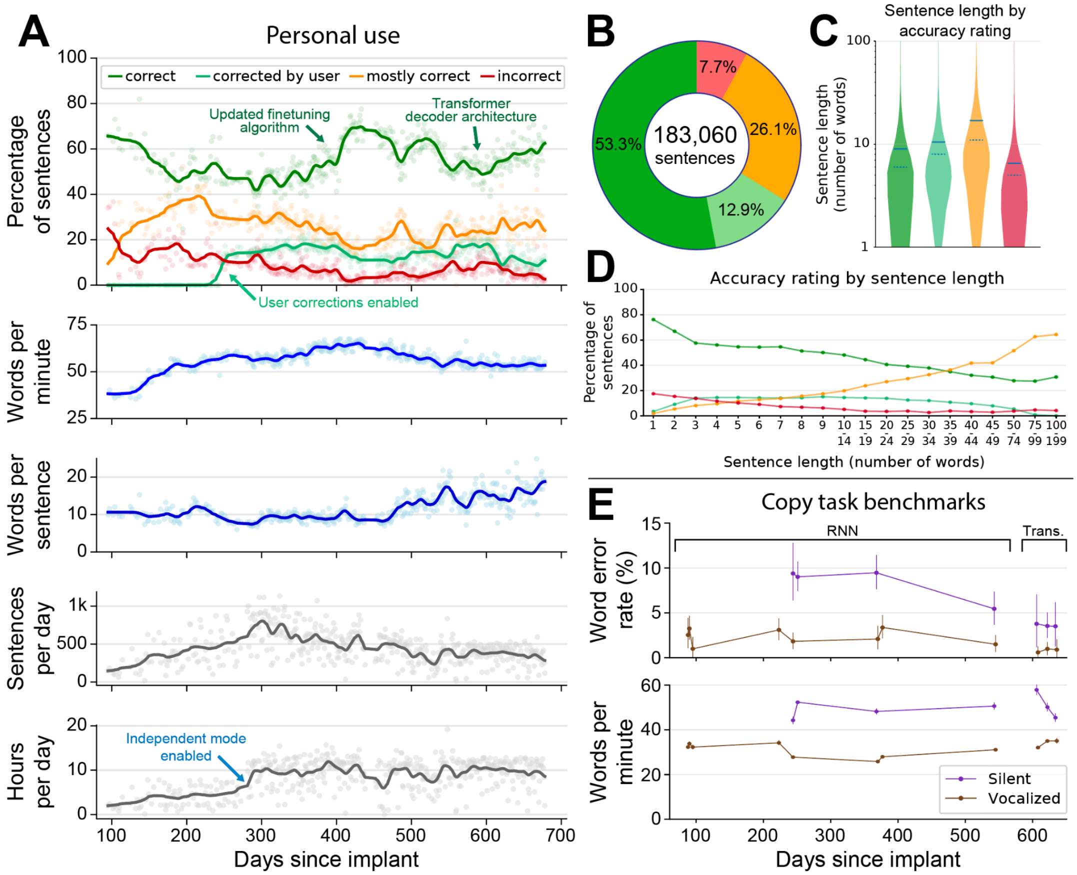

Figure 2. Speech decoding. (A) "Personal use" speech decoding usage and accuracy statistics by day. The first subplot shows the percentage of sentences that the participant reported to be (i) immediately correct, (ii) initially not correct but corrected through the user interface, (iii) mostly correct, or (iv) incorrect. Subplots 2-5 show the daily average rate of communication (words per minute), average words per sentence, total sentences communicated, and total hours of system use. To provide additional context, text and arrows on subplots indicate when substantive changes were made to the BCI system. **(B)** Distribution of self-reported sentence correctness among 183,060 sentences sourced from all personal use sessions. Color legend is the same as the top plot in panel **(A)**. All self-reported sentence accuracy ratings were made at the participant's discretion. **(C)** Cumulative distributions of sentence lengths as a function of participant-rated decoding accuracy. Solid horizontal blue lines are means, dashed lines are medians. **(D)** Sentence correctness rating probability displayed as a function of sentence length. **(E)** Word error rate (top) and speaking rate (bottom) in periodic Copy Task benchmark sessions using silent (violet) and vocalized (brown) speaking strategies. Vertical lines denote 95% confidence intervals. The final 3 benchmark sessions used the transformer-based decoder, all prior sessions used the RNN-based decoder.

8. Figure S5 - add units to spike band power in x-axis labels (or a.u. if arbitrary). Also, panels A and B titles both say "sentences". Does this mean that sentences were produced by the decoder while the participant was asleep? Please clarify

Thank you for pointing this out and for raising this question. We have added units for spike band power (μV²) to the figure, as suggested. The updated figure is shown below.

When T15 fell asleep while the speech decoder was enabled, the decoder *did* sporadically detect speech onset and decode nonsense “sentences”, which is why this figure is labeled as so. Given that this is indeed a bit of a silly/counter-intuitive use of the word, we’ve now added the scare quotes to the legend <Tired/Asleep “Sentences”>. As an aside, this very phenomenon motivated us to conduct a controlled research session wherein we recorded neural data while T15 was in various stages of wakefulness. The results of this experiment, shown in Figure S4A, demonstrate that neural data (mean spike band power) did change with T15’s alertness level. Figure S4 panels B-E then use detected speaking epochs during independent use, alongside eye tracking data to infer T15’s wakefulness state, to further examine how neural data and speech decoder confidence were affected by wakefulness.

We have edited the figure caption to better explain this:

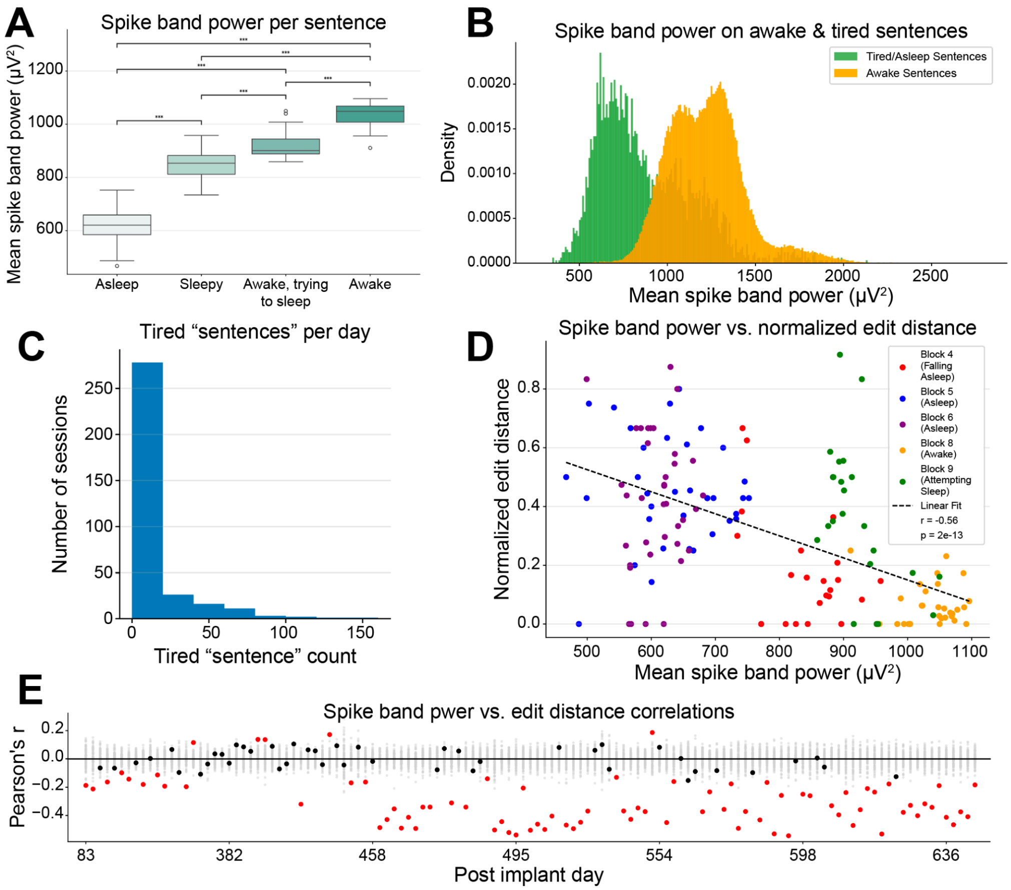

Figure S4. The participant's level of alertness affects neural signals and speech decoding accuracy. (A) Relationship between mean spike band power and putative alertness level. Neural data were recorded in a structured research session while the participant was observed to be asleep, sleepy, awake but trying to sleep, and awake. Spike band power varied significantly with alertness level (ANOVA with post hoc comparisons; ***p<0.001). **(B)** Distributions of spike band power during independent BCI use while the participant was alert versus tired/asleep. Each datum represents the mean spike band power for a sentence; when T15 was nominally asleep, the speech decoder occasionally falsely detected attempted speech and decoded "sentences", and we use those epochs alongside alert speaking epochs for analysis here. Data from all personal use sessions where eye tracking data was available are aggregated, resulting in 139,364 alert sentences and 3,714 tired/asleep "sentences". Alertness level was inferred using eye tracking data: if no eye tracking data was available for 2 minutes or more, alertness was categorized as tired/sleeping. **(C)** Number of daily "sentence" trials where the participant was tired or sleeping during independent use. 37.0% of days had at least 5 sentences that were categorized as tired. **(D)** Relationship between mean spike band power and speech decoding accuracy for one example day. Here, speech decoding accuracy is quantified as the edit distance between the predicted phoneme sequence output from the RNN/Transformer neural decoder and the predicted phoneme sequence output from the language model – higher values indicate worse speech decoding. Points are individual trials, colored according to the participant's estimated alertness level. A linear trendline was fit to the data, which had a significantly negative slope, indicating that speech decoding tended to be better on trials with higher average spike band power values which corresponded to when the participant was estimated to be more alert. **(E)** Linear correlation values between mean spike band powers and edit distance per trial across days of independent use that included 5 or more tired trials. Chance correlations were estimated with 100 shuffled datasets' values and shown in light gray. Significant correlations ($p < 0.01$) are marked in red.

9. How was asleep versus tired determined? Was there an objective measure of 'tired' or sleepiness?

We primarily used eye tracking data to infer the participant's wakefulness during independent BCI use. As discussed above, we observed that the speech decoder would sometimes sporadically detect speech while T15 was asleep or quite tired. By analyzing when speech was detected alongside eye gaze data, we could determine whether T15 was actually looking at the screen during detected speech epochs. If gaze was present during speech detection and if T15 used gaze to rate the accuracy of the decoded sentence, we knew that T15 was awake. If gaze was not detected during speech detection or for 2 minutes prior, and if decoded sentence accuracy was not rated, we could be quite confident that T15 was likely quite tired or not awake (as T15 almost always used eye gaze to control the BCI user interface while awake). We recognize that this is a simple heuristic and that it is relatively limited compared to an entire field of research focused on sleepiness and drowsiness that is beyond the scope of this study. Here,

we restrict ourselves to a very BCI-centric functional metric that could explain some of the variability in decoding performance.

We also conducted a controlled experiment wherein we recorded neural data while T15 was known to be awake, trying to fall asleep, or already asleep (Fig. S4A). This analysis highlighted a clear and significant difference in recorded neural data as a function of T15's wakefulness.

We have edited the caption of Figure S4 to include words like "putative alertness: and "nominally asleep" to signal that we do not have a gold-standard estimate of tiredness or sleep (e.g., no Polysomnography):

> **Figure S4. The participant's level of alertness affects neural signals and speech decoding accuracy. (A)** Relationship between mean spike band power and putative alertness level. Neural data were recorded in a structured research session while the participant was observed to be asleep, sleepy, awake but trying to sleep, and awake. Spike band power varied significantly with alertness level (ANOVA with post hoc comparisons; ***p<0.001). **(B)** Distributions of spike band power during independent BCI use while the participant was alert versus tired/asleep. Each datum represents the mean spike band power for a sentence; when T15 was nominally asleep, the speech decoder occasionally falsely detected attempted speech and decoded "sentences", and we use those epochs alongside alert speaking epochs for analysis here. Data from all personal use sessions where eye tracking data was available are aggregated, resulting in 139,364 alert sentences and 3,714 tired/asleep "sentences". Alertness level was inferred using eye tracking data: if no eye tracking data was available for 2 minutes or more, alertness was categorized as tired/sleeping. **(C)** Number of daily "sentence" trials where the participant was tired or sleeping during independent use. 37.0% of days had at least 5 sentences that were categorized as tired. **(D)** Relationship between mean spike band power and speech decoding accuracy for one example day. Here, speech decoding accuracy is quantified as the edit distance between the predicted phoneme sequence output from the RNN/Transformer neural decoder and the predicted phoneme sequence output from the language model – higher values indicate worse speech decoding. Points are individual trials, colored according to the participant's estimated alertness level. A linear trendline was fit to the data, which had a significantly negative slope, indicating that speech decoding tended to be better on trials with higher average spike band power values which corresponded to when the participant was estimated to be more alert. **(E)** Linear correlation values between mean spike band powers and edit distance per trial across days of independent use that included 5 or more tired trials. Chance correlations were estimated with 100 shuffled datasets' values and shown in light gray. Significant correlations ($p < 0.01$) are marked in red.

10. How accurate is the eye tracking data? Was it/would it be sustainably impacted by external factors (such as lighting conditions)?

We are using a Tobii Pro Spark for gaze tracking, which, according to the manufacturer's specifications, samples gaze at 60 Hz with a precision of 0.26° root mean square (RMS) and an accuracy of 0.45° RMS in optimal conditions. In practice, we found that the participant usually only needed to calibrate the eye tracker once at the start of each day. If he or the monitor dramatically shifted relative to one another throughout the day, another calibration may be required, which could easily be triggered through the user interface menu. When calibrated, T15 could reliably use the eye tracker to activate large on-screen buttons designed for this purpose, though the accuracy would likely be insufficient for pressing smaller buttons that are designed to be pressed by a computer mouse. Since we knew where the gaze-pressable buttons were on our user interface, we added an additional layer of software that "magnetized" gaze to nearby buttons, helping T15 press them more easily and consistently.

We did not observe dramatic impact on eye tracking accuracy by lighting conditions, though we note that T15's typical setup location is fairly consistently lit throughout the day as it is indoors and does not frequently have direct sunlight shining into nearby windows.

We have added some relevant details to the *Eye tracking* section of the methods:

> *Eye tracking*
> T15's eye gaze was tracked using a Tobii Pro Spark eye tracker (Tobii AB, Stockholm, Sweden) that was mounted at the bottom of the BCI system participant monitor. Calibration was performed at the start of each session (for both research sessions and independent sessions) and repeated as needed via an option available in the BCI system menu (Video 13). Gaze control typically required one calibration routine at the beginning of each day, plus potential additional recalibrations if T15 moved, if the monitor was moved, or if lighting conditions changed. The gaze calibration routine took less than 30 seconds and could be accessed at any time through the BCI user interface's menu screen. Gaze location was sampled at 60 Hz, averaged across both eyes, smoothed over time, and used to enable gaze-based selection of on-screen buttons. Button activation was triggered by maintaining gaze on an on-screen target for 0.5-1.0 seconds.

Reviewer #3 (Remarks to the Author):
This manuscript describes long-term (19 months) performance of combined speech and cursor decoding from a BrainGate2 clinical trial participant. The system demonstrated here maintained robust performance over more than two years, albeit with updates to the underlying decoding models. Crucially, the authors demonstrated that the clinical-trial participant could use the system with no assistance from researchers other than periodic updates to the decoders and software, requiring only assistance from a caregiver to connect the BCI and turn on the hardware, which usually required about 20 minutes. The participant used the system as his primary mode of communication and control of his computer to accomplish both professional and personal tasks. Cursor control required a total of only 10 minutes of calibration throughout the day. Interestingly, both speech and cursor/click control were decoded from the same neural signals recorded in speech motor cortex. Moreover, the authors present compelling data on the

stability of the neural signals over the course of the study. This report should be of great interest to a broad scientific audience. Although it does not present a major new scientific advance, it does make a substantial contribution to the burgeoning field of intracortical BCI's by demonstrating the feasibility, stability, and utility of long-term high performance multimodal intracortical BCI usage. This represents an important translational advance that is anticipated to have a significant impact on the field.

Major comments:
1. The manuscript could better distinguish the results of this work and those of the results from the authors' prior published works. This is not to diminish the novelty of this paper, which is certainly strong, but more an encouragement to clearly define what aspects of research have already been reported and have not, especially in the Introduction section. For speech decoding, if the authors reported the results from the same model and same system as reported in ref. [5], then the little-to-no daily calibration and high decoding accuracy aspect have already been published. The authors should consider clarifying that the novelty comes from long-term usage and results, as well as multi-modal cursor and speech decoding.

Thank you for this suggestion. Indeed as the reviewer pointed out, we have previously reported both brain-to-text speech decoding [Card et al. 2024, *NEJM*] and cursor control [Singer-Clark et al. 2025, *JNE*] with the same participant.

[Card et al. 2024, *NEJM*] demonstrated high-accuracy speech decoding (2.5% word error rate) in prompted research tasks and for personal communication with researcher assistance. The present study extends this work by (1) enabling independent use of the BCI without researcher assistance, (2) extending the included data from post-implant day 244 (as in [Card et al. 2024, *NEJM*]) to post-implant day 678 (thus more than doubling the longest-ever reported use of a speech BCI), (3) introducing a new transformer-based phoneme encoder with better accuracy (<1% word error rate) and less required calibration, and (4) refining the personal use BCI system and adding a variety of user-requested features.

[Singer-Clark et al. 2025, *JNE*] demonstrated cursor decoding with a linear decoder in research tasks and for personal computer control in research sessions. The present study extends this work by (1) introducing a new RNN-based cursor decoder that offers more stable decoding and lower calibration requirements, (2) integrates the cursor training and inference pipeline into the personal use BCI system, and (3) demonstrates that this cursor control pipeline can be used independently and on a daily basis.

We have added text to the Introduction to briefly summarize the advancements offered by this study beyond previous studies:

> Here, we report the long-term, independent use of a multimodal intracortical BCI that enables both brain-to-text speech and computer cursor control. This study builds upon the core brain-to-text decoding capability of [5] and ventral motor cortex cursor control of [18], and adds new decoding architectures with higher accuracy and stability, thousands of

hours of independent BCI usage over several months, and a refined BCI user interface with additional features that enhance the efficacy of independent communication and digital access.

With care partner assistance largely restricted to donning and doffing the hardware and initializing the software, a man with paralysis and severe dysarthria due to ALS used the system in his home nearly every day for 19 months, accumulating over 3,800 hours of independent use. The system incorporated novel decoding architectures for both speech and cursor control, as well as multiple software features that enabled independent use and continuous decoder finetuning. For speech, we developed a transformer-based brain-to-text decoder that outperformed prior RNN-based models[5], requiring little-to-no daily calibration and achieving a new state-of-the-art word accuracy of 99.2% in a prompted word Copy Task (125,000 word vocabulary). For cursor control, we implemented an RNN-based decoder that matched or exceeded the performance of linear models, also with reduced calibration requirements. Using this system, the participant communicated more than 180,000 sentences at conversational speeds and used the speech and cursor decoders together to independently operate his personal computer – sending messages, browsing the internet, participating in video calls, and maintaining full-time employment – despite being paralyzed.

2. Was the shift from the original RNN-based model to transformer on Day 600 a sudden switch or was the participant given an opportunity to practice and adapt to the new model? It may be surprising that the shift to a completely new model did not cause any temporary drop in performance. If this is true, can the authors offer any explanation, however hypothetical, as to why?

Thank you for this insightful question. Although we did not perform studies of back-and-forth model switches to rigorously test whether changing brain-to-text phoneme encoder models led to a short-term performance change, we did not observe any temporary drop in performance during the transition from the RNN-based phoneme encoder to the new transformer-based phoneme encoder. We believe that this lack of transition-related performance dip is due to a variety of reasons.

First, we note that the participant maintained the same attempted speaking strategy throughout this transition. Unlike tightly closed-loop tasks like cursor control [Gilja*, Nuyujukian* et al. Nature Neuroscience 2012; Stavisky et al. JNE 2015; Pandarinath et al. eLife 2017] where different decoders might have changes in their immediate dynamics, brain-to-text feedback is delayed by 160-240 milliseconds during attempted speech (plus the final rescoring at the end of each sentence), making it less of an active sensorimotor control task with millisecond by millisecond corrections based on on-screen feedback. Due to this quasi open-loop nature, we did not believe it necessary to add a training period when planning decoder changes in the study.

Second, we note that before the new transformer-based phoneme encoder model was used for independent communication by the participant, it was extensively validated in offline analyses and in prompted sentence research sessions (Fig. 4E). Finally, we note that the language model pipeline that converted the encoder's predicted phoneme probabilities into words remained unchanged during the transition from the RNN-based phoneme encoder to the new transformer-based phoneme encoder.

We have edited the Methods section text to address this point:

> <u>Transformer decoder architecture and pre-training</u>
> On post-implant day 600, we switched from using the RNN-based decoder to a more accurate transformer-based decoder. We did not perform formal multiple-repetitions evaluation of the effect of switching decoder architectures. However, we did not observe any temporary reduction in decoding performance (Fig. 2A, 2E), which is consistent with the quasi-open loop nature of the brain-to-text BCI.
>
> The transformer-based decoder consisted of three stages: day-specific transformation, input striding, and a 12-block causal transformer with multi-head attention. Day-specific embedding input layers added a learned day-specific vector to each input timestep to correct for inter-day variability. This linear offset (as opposed to a nonlinear transformation as was used for the RNN[5,7]) was empirically found to perform better for this architecture. The transformer decoder applied multi-headed causal attention with Rotary Positional Embeddings[27] and used RMSNorm[28] for normalizing hidden states. Outputs from the transformer were fed through a feedforward layer to produce phoneme probability distributions every 80 ms. Like the RNN, this model was also trained with CTC loss, which automatically aligned neural data and labeled phoneme sequences.
>
> Offline model pre-training used all previously recorded labelled data (i.e. Copy Task trials and personal use trials that were confirmed by the participant to be decoded correctly), excluding trials that had excessive electrical artifacts or were too long, with a 90/10 train/validation split per day. Data were augmented with noise and constant offsets, and then smoothed using a Gaussian kernel (σ = 40 ms). Mixed-day batches (6 random days of data, 16 randomly-selected trials from each) were used to regularize learning. Optimization was performed with AdamW using a cosine decay schedule and warmup. Offline model pre-training lasted for 24-36 hours (400,000-600,000) batches and was run on a single RTX 4090 GPU.

3. The calibration time described in the cursor decoding session for both the old and new model seem to be substantial enough for the participant to have noticed. Is the continued calibration throughout the day initiated by the user or the decoding pipeline?

The participant did state that they noticed the difference in calibration requirements between the linear cursor decoder and the RNN-based cursor decoder. Cursor calibration only occurred

when the participant chose to enter the cursor calibration routine (i.e., the cursor decoder was not continuously finetuned in the background like the speech decoder was). So, when the participant noticed that cursor control was degrading, he may choose to enter the calibration routine to improve his control.

We have clarified this point in the Methods:

> *Cursor decoding*
> Calibration task
> The cursor calibration task could be initiated at any time through the user interface menu. Cursor decoders were only updated during this calibration task and did not undergo background finetuning. Until post-implant day 621, the calibration task was a center-out-and-back eight target "Radial 8" acquisition task with fixed target distance (40% of screen height), fixed target radius (5% of screen height), and the next target predictably in the center after every outward trial[17]. After day 621, the task was made more varied and engaging to elicit more diverse cursor movements (similar to the previously reported Fitts task [29]). Target positions could be anywhere in the 2-D workspace (1920 x 1080 pixels), leading to both shorter and longer target distances, and cursor trajectories at any angle. Target radii were smaller than in the Radial 8 task and varied (2.5-4.0% of screen height).

4. Figure 4C. Day 25 modulation map for 55b array looks very different from the ones later on. Can the authors explain why? Also, how did the authors choose the four dates? They are not evenly spaced.

Thank you for this observation and question. The table below shows the cosine similarity values between the neural modulation for each array on post-implant days displayed in Fig.4C (post-implant days 242, 460, and 678). These values are consistent with the population cosine similarity values shown in Fig. 4D. So, although post-implant day 25 might qualitatively look a bit different, the aggregate results in Figure 4d indicate that overall array 55b's tuning was quantitatively similarly stable to the arrays in areas v6v, and 4.

**Array**	**Comparison Days**					
	**25 vs 242 (217 day gap)**	**242 vs 460 (218 day gap)**	**460 vs 678 (218 day gap)**	**25 vs 460 (435 day gap)**	**242 vs 678 (436 day gap)**	**25 vs 678 (653 day gap)**
55b	0.782	0.785	0.919	0.743	0.793	0.756
d6v	0.128	0.330	0.138	0.091	0.038	-0.051
M1	0.733	0.751	0.888	0.701	0.746	0.661

v6v	0.814	0.797	0.929	0.753	0.830	0.782

The presented days of data initially used in Figure 4C were chosen to mostly-evenly span the period from our oldest day of data (post-implant day 25) to the most recent (post-implant day 678). In response to a separate reviewer comment (Reviewer 1, minor issue 6), we have adjusted this figure to span this period more evenly in a uniform manner. Each of the four presented days are now separated by 217 or 218 days from their neighbors. We have also added this information to the caption.

Here is the updated figure and caption:

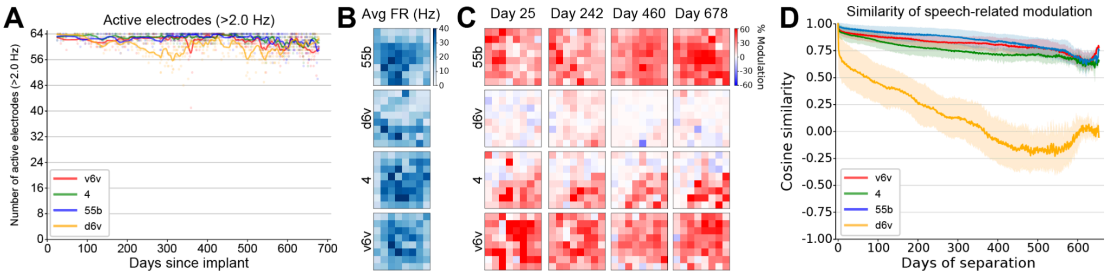

Figure 4. Neural stability. (A) Number of electrodes per array with firing rates of 2 Hz or greater during speaking epochs, plotted by post-implant day. Dots are individual data points and lines are Gaussian-smoothed approximations. Colors correspond to the four arrays. **(B)** Average firing rate for each electrode during speaking epochs from the entire 444-session dataset. Electrode locations are drawn according to their physical location on each of the 4 arrays, where up corresponds to dorsal and left corresponds to rostral. **(C)** Speech-related neural modulation for each electrode on a given example day, expressed as the percentage change in spike band power between rest epochs and speaking epochs. Each column corresponds to the post-implant day indicated by the column title. Days are chosen to uniformly span the post-implant period (25-678 days post-implant). **(D)** Cosine similarity between daily speech-related neural modulation vectors, as a function of the number of days between each pair. Colors correspond to the four arrays. Solid lines are means and shaded regions represent one standard deviation around the mean.

5. I think it would be beneficial to also include impedance measurements in the neural signal stability section.

Thank you for this suggestion. We have added a supplemental figure (Fig. S6) that shows impedance values over time (also pasted in this document, below), and we now reference and discuss these results in the *Neural signal stability* results section. Please note that impedance values were not recorded on all days, but we have included the data that are available.

We have edited the Methods text to include a reference to the new supplemental figure:

Neural signal stability
Speech and cursor decoding performance remained accurate for more than 19 months post-implant, suggesting that the underlying neural signals retained sufficient quality to support high-accuracy decoding. To directly examine the stability of these signals, we analyzed neural activity across the 678-day post-implant period.

Multi-unit action potentials were consistently detected on nearly all electrodes throughout the study. Over 90% of electrodes on each 64-electrode array could reliably detect spiking activity at 2 Hz or higher throughout more than 19-months after implant (Fig. 4A). The dorsal 6v array occasionally exhibited fewer active spiking electrodes than the other arrays during speaking epochs, consistent with its lower tuning for speech[5] and stronger tuning for cursor control[18]. Average firing rates for each electrode during speech epochs across the entire dataset are shown in Figure 4B. Impedance values for each microelectrode on each array were measured on many (but not all) sessions (Fig. S6).

To assess the stability of task-relevant neural modulation, we measured the change in spike band power between rest and speech epochs for each electrode on each day. These speech modulation maps appeared qualitatively stable across hundreds of days (Fig. 4C). We quantified this by computing the cosine similarity between daily neural modulation vectors for each array as a function of time separation (Fig. 4D). Cosine similarities for all arrays – except the d6v array, which did not exhibit much speech-related modulation – remained above 0.6 even for comparisons separated by more than 18 months, demonstrating that task-relevant neural representations remained quite stable for at least 19 months.

And this is the new supplemental figure, which shows a slow trend of lower impedances consistent with Utah arrays in other humans (e.g., https://iopscience.iop.org/article/10.1088/1741-2552/ac1add#jneac1addf3; https://pmc.ncbi.nlm.nih.gov/articles/PMC8500669/), which need not correspond to decreased decoding accuracy (our consortium has a preprint on this topic including 20 arrays across 14 participants: https://www.medrxiv.org/content/10.1101/2025.07.02.25330310v1) :

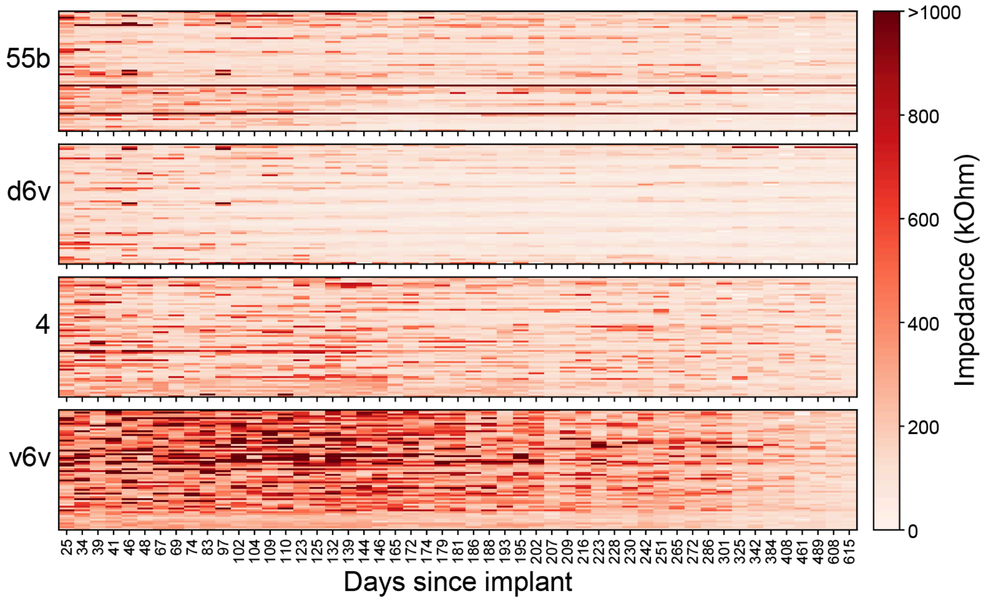

Figure S6. Impedance values over time. Impedance values for each microelectrode on each array were regularly measured through the Blackrock Central software interface. Impedance values for each electrode over time are shown here, grouped by array, according to the color bar.

6. For the click decoder, since the participant could adjust the sensitivity, what is the average T throughout the reporting period and its distribution?

Thank you for noting that this click decoder detail was not previously available to the reader. In response, we have added a new supplemental figure (Fig. S8) that explores both the requested click sensitivity and cursor speed, since T15 could adjust both of these via the BCI system's user interface. For these analyses, we analyzed 126 days of personal use sessions (1208.2 total hours) where T15 used the neural cursor. Note that we excluded sessions where T15 did not use neural cursor, and we also excluded early sessions where click sensitivity or cursor speed were not tracked.

The default click sensitivity value upon starting the BCI system was 0.2, and T15 could increase this up and down in increments of 0.2 (bounded between 0 and 1). He often increased it once (from 0.2 to 0.4) at the start of neural cursor usage, and only occasionally changed it throughout the day afterward. The distribution of click sensitivities across 1208.2 hours of BCI usage is shown in Fig. S8A, below. Across all data, the average click sensitivity was set to 0.36 ± 0.12 (mean ± standard deviation).

Cursor speed initialized to 0.40 upon starting the BCI system and T15 could adjust it in 0.05 increments (bounded between 0 and 1). The distribution of cursor speeds across 1208.2 hours of BCI usage is shown in Fig. S8B, below. Across all data, the average cursor speed was set to 0.33 ± 0.05.

This is the new supplemental figure and caption:

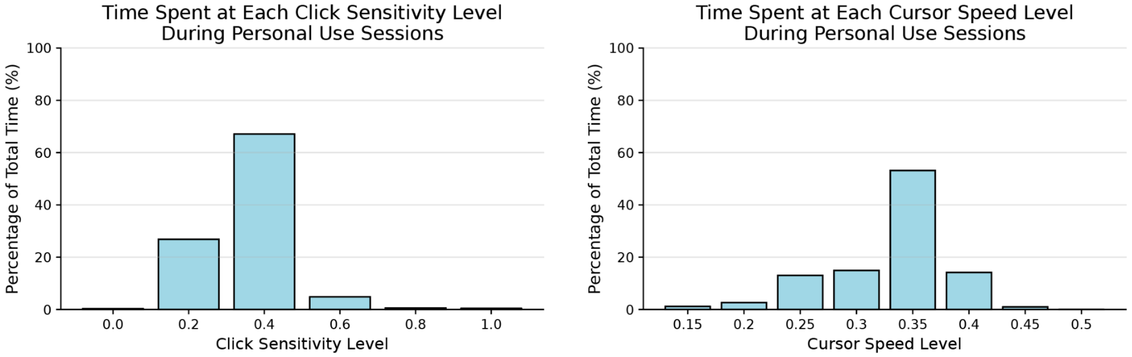

Figure S8. Neural click sensitivity and neural cursor speed settings. T15 could adjust the neural click sensitivity and the neural cursor speed within the BCI user interface. Data shown is from 126 days (1208.2 hours) of personal use wherein T15 used the neural cursor and the click sensitivity and cursor speed metrics were tracked. **(A)** Distribution of click sensitivity values. Click sensitivity initiated to 0.20 when the BCI system was initialized, and could be adjusted up and down in increments of 0.20 (bounded between 0 and 1). Across all data, the average click sensitivity was set to 0.36 ± 0.12 (mean ± standard deviation). **(B)** Distribution of cursor speed values. Cursor speed initiated to 0.40 when the BCI system was initialized, and could be adjusted up and down in increments of 0.05 (bounded between 0 and 1). Across all data, the average cursor speed was set to 0.33 ± 0.05.

7. Given the fact that the decoding accuracy evaluation was based on self-reports, did the authors attempt to measure accuracy from self-ratings?

We informed the participant that the speech decoder's accuracy could degrade if he marked incorrectly decoded sentences as correct, as the model was finetuned based on his ratings. Additionally, we routinely checked in with the participant to ensure that he understood the rating system and was using it as intended. He did note that he sometimes (rarely) pressed the wrong accuracy rating button by accident, but he estimated that this happened on less than 1% of sentences. We also worked with him to optimize the user interface button sizes, press speeds, and gaze magnetizations to minimize accidental presses.

We have added a sentence to the Methods to address this:

> During independent BCI use, we did not know the ground truth of what T15 was saying, and hence we could not compute the true phoneme and word error rates. Instead, we asked the participant to use gaze or cursor control to label the accuracy of each decoded utterance as "100% correct", "one word wrong", "mostly correct", or "incorrect". He also had the opportunity to make corrections to decoded sentences before rating the

accuracy. We instructed T15 on the importance of proper labeling of the sentences and encouraged him not to under- or over-report the decoded outcome. We shared with him that the proportion of “correct” sentences would be further used for the recalibration that was necessary for ongoing system maintenance, and that incorrect sentences would be used to help troubleshoot our system for future software iterations. Hence, he was incentivized to correctly label outcomes to maintain accurate ongoing use. T15 told us that he believed that he pressed the wrong accuracy rating button by accident on less than 1% of sentences, although we note that we did not have a way to independently check the ground truth of independent use sentences. When asked how he chose between labeling a sentence with multiple incorrectly decoded words as “mostly correct” or “incorrect”, T15 told us that he uses a threshold of 2/3rds (Table S1): sentences with more than 2/3rds of words decoded correctly are “mostly correct”, and sentences with less than 2/3rds of words decoded correctly are “incorrect”.

Beyond the above efforts to help the participant be maximally accurate in his self-reports, we are unsure how to objectively measure accuracy of self-ratings, given that we do not have ground truth labels for his independently communicated sentences, and we can not reasonably ask him what he meant to say on all imperfectly decoded sentences.

Minor comments:
1. At least one other study (Vansteensel et al. 2016) has reported independent home use of a BCI by a participant with ALS. I believe the same authors also reported their signal stability over multiple years.

Thank you for highlighting this prior work. We have added a citation and brief discussion of this study (and the related Vansteensel et al. 2024, *NEJM*) to the Discussion, while noting that it used a different sensor technology (ECoG) and a different communication scheme (click):

This study demonstrates that an intracortical brain-computer interface can support long-term, independent communication and computer control by a person with severe paralysis and dysarthria due to ALS. Over a 19-month period, the participant used the system in his home for more than 3,800 hours, speaking over 183,000 sentences, browsing the internet, managing personal and professional communications, and maintaining full-time employment. These outcomes establish that a single, speech motor cortex BCI can provide practical, multimodal assistive technology for daily life – enabling both speech and cursor control with high performance and without researcher supervision.

This work successfully addresses two critical gaps that have limited the clinical viability of high-performance intracortical BCIs: the need for researcher-free, at-home operation and the need for sustained decoding performance over long durations. While previous studies have demonstrated high speech or cursor control accuracy in controlled settings, few have supported independent daily use in a home environment and none to our

knowledge have done so for speech or for both speech and computer control simultaneously. Prior work with electrocorticographic (ECoG) BCIs has shown that independent, at-home use is achievable[24,25], though these systems relied on click-based decoding, which offers slower communication rates than attempted speech. Whether multielectrode array-based BCIs could support independent and stable long-term use remained previously unknown. Here, the participant and his care partners were able to operate the system independently after an initial training period, and he continued to use it almost every day over nearly two years (444/653 days overall, 364/397 days after independent use was enabled).

2. Figure 1B. What is the gesture being used in the click decoder?

T15 was initially instructed to attempt to squeeze his right hand for the click, although he noted that over time he stopped thinking about squeezing his hand and instead relied on "intuition" to simply try and click. We have clarified this in the text:

Click decoding
Click decoding used a linear decoder as reported previously[18], which T15 calibrated anew each day as part of the same tasks used to calibrate cursor control. We initially instructed T15 to attempt to squeeze his right fist for a click, though he noted that over time he stopped thinking about squeezing his hand and instead relied on intuition to simply try and click[17]. The decoder's weights were computed online using logistic regression every 3.0 seconds during calibration.

Similarly, we now also include the instructed imagery for neural cursor control in the relevant Methods text, which appears prior to the section about Click decoding:

Cursor decoding
Imagery
During neural cursor control, we initially instructed T15 to attempt to move his right arm and hand as if he were controlling a computer mouse, though he noted that over time he stopped thinking about moving and instead relied on "intuition" to simply move the cursor[17].

Calibration task
The cursor calibration task could be initiated at any time through the user interface menu. Cursor decoders were only updated during this calibration task and did not undergo background finetuning. Until post-implant day 621, the calibration task was a center-out-and-back eight target "Radial 8" acquisition task with fixed target distance (40% of screen height), fixed target radius (5% of screen height), and the next target predictably in the center after every outward trial[17]. After day 621, the task was made more varied and engaging to elicit more diverse cursor movements (similar to the previously reported Fitts task[29]). Target positions could be anywhere in the 2-D

workspace (1920 x 1080 pixels), leading to both shorter and longer target distances, and cursor trajectories at any angle. Target radii were smaller than in the Radial 8 task and varied (2.5-4.0% of screen height).

3. Figure 1E. What is the Other category?

In Figure 1E, “other” just refers to time where the BCI system was on, but the participant was not doing anything with it – not speaking, not using cursor, and not using gaze to navigate the user interface. This might include times when he is reading an article or watching a video on his personal computer, for example. We now clarify this in the figure caption:

> **Figure 1. Independent use of the multimodal intracortical BCI. (A)** Schematic of the participant using the multimodal intracortical BCI to control their personal computer. As the participant tried to speak, his neural activity was decoded into words on a screen. He could also control his computer by attempting to move or squeeze his hand. The system also integrated eye tracking to enable the participant to select on-screen buttons by looking at them for a short time. The participant could switch between gaze-based and neural cursor-based control of the user interface by selecting his preference through the custom-made GUI. **(B)** Locations of four 64-microelectrode arrays in the participant’s dominant ventral precentral gyrus (left). The dotted line is the dominant central sulcus. Each electrode records activity from individual neurons and local field potentials. Recorded neural signals were processed into binned sequences of neural features (middle) and inputted to speech, cursor, or gesture decoders to decode the user’s intended words, cursor movements, or clicks (right). **(C)** User interface examples during speech decoding (left), sentence rating (middle), and sentence correction (right). **(D)** Cumulative hours of system usage over time. Independent use began at post-implant day 281. **(E)** Time distribution of how the system was used across 3,801 hours sourced from all personal use sessions. “Other” refers to idle time where the BCI system was active but the participant was not using it.

4. Some of the imprecise terms could be replaced or followed by concrete numbers if the information is not otherwise provided in the manuscript. Examples include:
‘…used the system in his home [nearly every day] for 19 months…’ What is the exact frequency?
‘requiring [little-to-no] daily calibration’ What does ‘little’ mean here?

Thank you for pointing out that these terms could be more precise. We have edited these imprecise terms accordingly:

In the Discussion:

> This work successfully addresses two critical gaps that have limited the clinical viability of high-performance intracortical BCIs: the need for researcher-free, at-home operation

and the need for sustained decoding performance over long durations. While previous studies have demonstrated high speech or cursor control accuracy in controlled settings, few have supported independent daily use in a home environment, and none to our knowledge have done so for speech or for both speech and computer control simultaneously. Prior work with electrocorticographic (ECoG) BCIs has shown that independent, at-home use is achievable[24,25], though these systems relied on click-based decoding, which offers slower communication rates than attempted speech. Whether multielectrode array-based BCIs could support independent and stable long-term use remained previously unknown. Here, the participant and his care partners were able to operate the system independently after an initial training period, and he continued to use it almost every day over nearly two years (444/653 days overall, 364/397 days after independent use was enabled).

The system's stability and usability were made possible by several advances. First, we developed improved architectures for both speech and cursor decoding that reduced calibration time and increased robustness. The speech decoder, built on a transformer-based model, achieved state-of-the-art performance (99.2% word accuracy in Copy Tasks, 125,000 word vocabulary) while requiring little or no daily explicit recalibration from the user (0-20 prompted calibration sentences at the start of each day at the user's discretion). The speech decoder's computational expressiveness[26] and continuous finetuning also helped accommodate neural nonstationarities that have challenged earlier BCI systems with lower channel counts and less flexible algorithms[27]. The cursor decoder, upgraded from a linear model to an RNN, similarly reduced calibration time while preserving or improving performance. Both modalities were decoded from the same neural activity recorded in speech motor cortex, where we found sufficient tuning for both speech-articulatory and hand motor imagery. Second, we implemented software features – such as background decoder calibration, gaze-based toggles, and on-screen rating and correction tools – that allowed the system to adapt to the participant's needs over time, including his shift from vocalized to silent speech. Finally, we automated the system startup and shutdown so that trained care partners could don and doff the system with a few simple steps. We also optimized the system so that, once running, it could operate continuously for up to 19 hours without any care partner intervention.

5. Figure 2 B and C: It might make sense to use Day 600 as another cutoff point since a new model was used. It could be beneficial to add two more arrows to Figure 2A indicating Day 412 and Day 600.

In response to a separate reviewer comment, we have removed the additional pie chart (formerly Fig. 4C) that showed sentence accuracy ratings after day 412 to make room for the now-included sentence length vs. accuracy plots. However, we have taken your suggestion to add additional arrows to Figure 2A indicating day 412 and day 600:

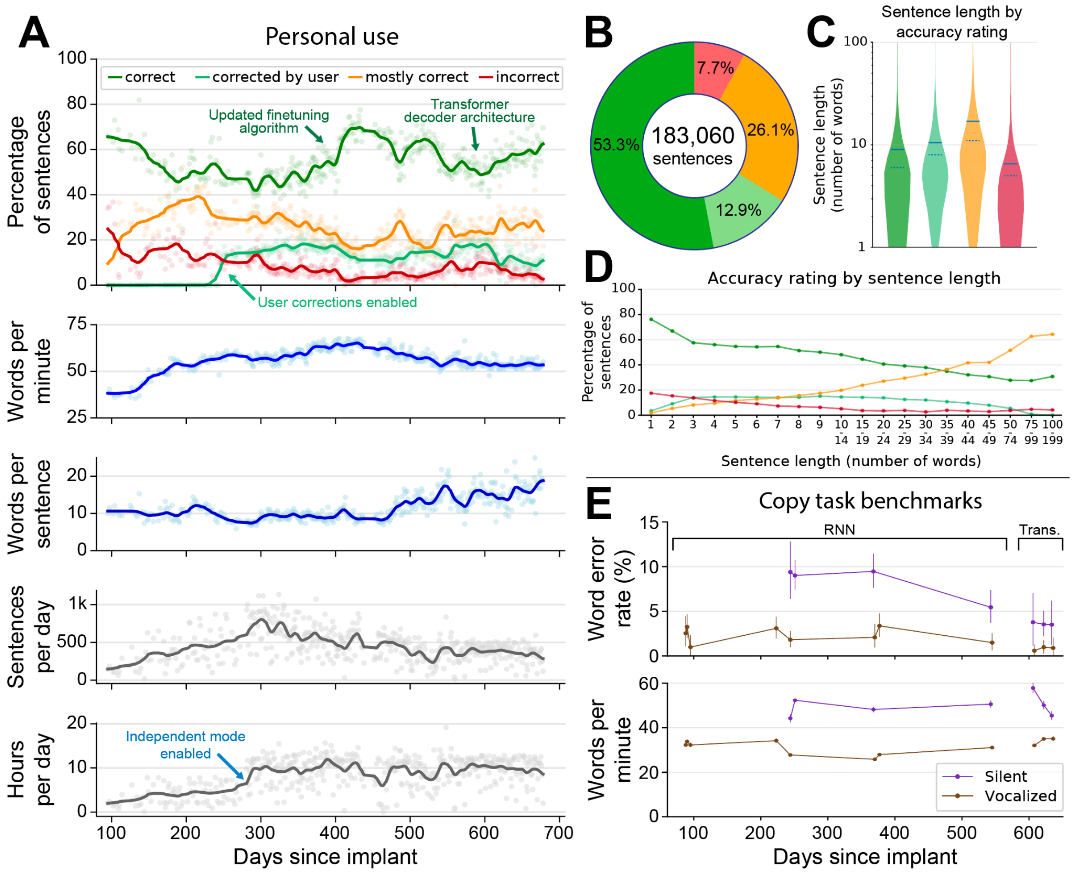

Figure 2. Speech decoding. (A) "Personal use" speech decoding usage and accuracy statistics by day. The first subplot shows the percentage of sentences that the participant reported to be (i) immediately correct, (ii) initially not correct but corrected through the user interface, (iii) mostly correct, or (iv) incorrect. Subplots 2-5 show the daily average rate of communication (words per minute), average words per sentence, total sentences communicated, and total hours of system use. To provide additional context, text and arrows on subplots indicate when substantive changes were made to the BCI system. **(B)** Distribution of self-reported sentence correctness among 183,060 sentences sourced from all personal use sessions. Color legend is the same as the top plot in panel **(A)**. All self-reported sentence accuracy ratings were made at the participant's discretion. **(C)** Cumulative distributions of sentence lengths as a function of participant-rated decoding accuracy. Solid horizontal blue lines are means, dashed lines are medians. **(D)** Sentence correctness rating probability displayed as a function of sentence length. **(E)** Word error rate (top) and speaking rate (bottom) in periodic Copy Task benchmark sessions using silent (violet) and vocalized (brown) speaking strategies. Vertical lines denote 95% confidence intervals. The final 3 benchmark sessions used the transformer-based decoder, all prior sessions used the RNN-based decoder.

6. I think some context is missing in the neural signal stability section. It would be nice if the authors could provide this context and explain (or theorize) how this study was able to overcome presumed instability.

The reviewer is correct that there is an existing concern that chronic intracortical microelectrode arrays exhibit large amounts of instability over time, either due to neural signal degradation or nonstationarities over time due to array micromotion or autoimmune responses. This issue may have been previously exacerbated for intracortical BCIs by neural decoding algorithms that were trained on small amounts of data and/or could not handle instability well (e.g., linear cursor decoders). It could also be exacerbated by low recording channel counts, where any small change in neural signals affects a larger proportion of the total recorded neural population.

However, we argue that our results demonstrate that chronic intracortical microelectrode array signals can be quite stable on the order of weeks, as they are for our participant (Fig. 4). Higher channel counts compared to previous studies (256 here, 128 or 96 or in most previous studies) may also contribute to enhanced decoding stability. Finally, our speech decoder is specifically engineered to be trained on large amounts of data, is highly computationally expressive in order to be able to handle nonstationary input signals (which our early work in a macaque pre-clinical model showed can address non-stationarities in Utah array records [Sussillo*, Stavisky*, Kao* et al. Nature Communications 2016]), and is constantly finetuned to adjust to slowly shifting neural representations.

We have added some text to the Discussion section to address this point:

> The system's stability and usability were made possible by several advances. First, we developed improved architectures for both speech and cursor decoding that reduced calibration time and increased robustness. The speech decoder, built on a transformer-based model, achieved state-of-the-art performance (99.2% word accuracy in Copy Tasks, 125,000 word vocabulary) while requiring little or no daily explicit recalibration from the user (0-20 prompted calibration sentences at the start of each day at the user's discretion). The speech decoder's computational expressiveness[26] and continuous finetuning also helped accommodate neural nonstationarities that have challenged earlier BCI systems with lower channel counts and less flexible algorithms[27]. The cursor decoder, upgraded from a linear model to an RNN, similarly reduced calibration time while preserving or improving performance. Both modalities were decoded from the same neural activity recorded in speech motor cortex, where we found sufficient tuning for both speech-articulatory and hand motor imagery. Second, we implemented software features – such as background decoder calibration, gaze-based toggles, and on-screen rating and correction tools – that allowed the system to adapt to the participant's needs over time, including his shift from vocalized to silent speech. Finally, we automated the system startup and shutdown so that trained care partners could don and doff the system with a few simple steps. We also optimized the system so that, once running, it could operate continuously for up to 19 hours without any care partner intervention.

7. It does seem like the overall operation of the system still requires a small dependency on eye tracking. If this is indeed the case, the authors should make this clearer.

Neural cursor control could be used throughout the entire BCI user interface as a control scheme, as shown in the newly-included Video 11. However, T15 preferred to use gaze control in the BCI user interface while reserving the neural cursor to control his personal computer.

We have updated the Figure 1 caption text to explain this:

> **Figure 1. Independent use of the multimodal intracortical BCI.** (A) Schematic of the participant using the multimodal intracortical BCI to control their personal computer. As the participant tried to speak, his neural activity was decoded into words on a screen. He could also control his computer by attempting to move or squeeze his hand. The system also integrated eye tracking to enable the participant to select on-screen buttons by looking at them for a short time. The participant could switch between gaze-based and neural cursor-based control of the user interface by selecting his preference through the custom-made GUI. (B) Locations of four 64-microelectrode arrays in the participant's dominant ventral precentral gyrus (left). The dotted line is the dominant central sulcus. Each electrode records activity from individual neurons and local field potentials. Recorded neural signals were processed into binned sequences of neural features (middle) and inputted to speech, cursor, or gesture decoders to decode the user's intended words, cursor movements, or clicks (right). (C) User interface examples during speech decoding (left), sentence rating (middle), and sentence correction (right). (D) Cumulative hours of system usage over time. Independent use began at post-implant day 281. (E) Time distribution of how the system was used across 3,801 hours sourced from all personal use sessions. "Other" refers to idle time where the BCI system was active but the participant was not using it.

And in the *Cursor decoding* subsection of the Results:

> T15 configured and toggled cursor control through the BCI's gaze-based interface, which allowed him to select on-screen buttons to start or stop control, paste recent decoded sentences, and issue function key presses (e.g., escape key; Fig. S6). He could also use the neural cursor to navigate the BCI user interface (Video 11), though he typically stuck to gaze-based control there while reserving the neural cursor for his personal computer. Each day, he calibrated the cursor and click decoders via a short calibration routine[17,18]. For calibration we used a center-out-and-back target acquisition task with fixed target distance and size until day 621, after which we used a more difficult task with varied target positions and smaller target sizes (see Methods).
